# Self-Assembled Molecules for Hole-Selective Electrodes in Highly Stable and Efficient Inverted Perovskite Solar Cells with Ultralow Energy Loss

**DOI:** 10.1021/acsaem.2c02880

**Published:** 2023-01-13

**Authors:** Wenhui Li, Michele Cariello, Maria Méndez, Graeme Cooke, Emilio Palomares

**Affiliations:** † Institute of Chemical Research of Catalonia (ICIQ-BIST), Avda. Països Catalans, 16, 43007Tarragona, Spain; ‡ School of Chemistry, University of Glasgow, GlasgowG12 8QQ, U.K.; § Catalan Institution for Research and Advanced Studies (ICREA), 08010Barcelona, Spain

**Keywords:** self-assembled monolayers, hole-selective contacts, ultralow energy loss, inverted perovskite solar cells, long-term stability

## Abstract

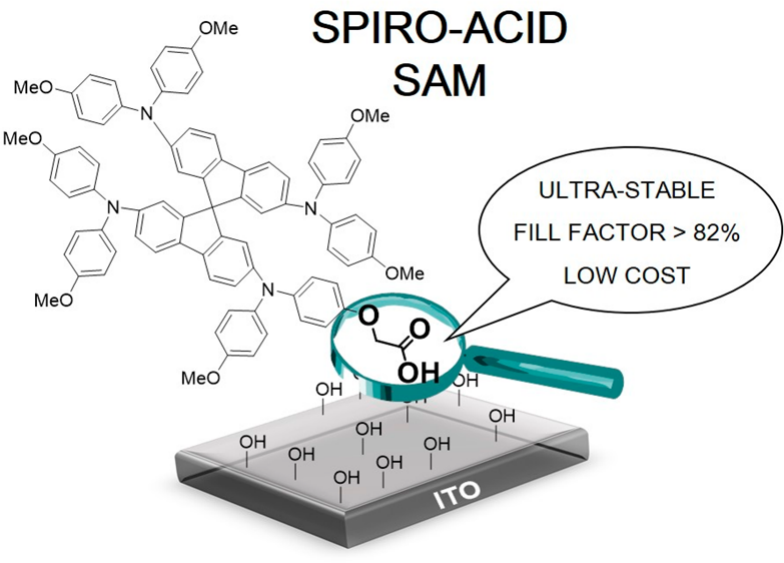

Good selective contacts are necessary for solar cells that are efficient and have long-term stability. Since 1998, with the advent of solid-state dye sensitized solar cells (DSSC), **Spiro-OMeTAD** has become the reference hole-transporting material. Yet, for efficient solar cells **Spiro-OMeTAD** must be partially oxidized with chemical dopants, which compromises the long-term stability of the solar cell. Alternatively, semiconductor polymers such as **PTAA** have been also studied, matching or improving the solar cell characteristics. However, **PTAA**-based devices lack long-term stability. Moreover, both **Spiro-OMeTAD** and **PTAA** are expensive materials to synthesize. Hence, approaches toward increasing the solar cell stability without compromising the device efficiency and decreasing the manufacturing cost are very desirable. In this work we have modified **Spiro-OMeTAD**, by an easy-to-use methodology, by introducing a carboxylic acid anchoring group (**Spiro-Acid**), thereby allowing the formation of self-assembled monolayers (SAMs) of the hole-transporting material in dopant-free p–i–n hybrid perovskite solar cells (iPSCs). The resulting device showed a champion efficiency of 18.15% with ultralow energy loss, which is the highest efficiency among **Spiro-OMeTAD**-based iPSCs, and a remarkable fill factor of over 82%, as well as excellent long-term illumination stability. Charge transfer and charge carrier dynamics are studied by using advanced transient techniques to understand the interfacial kinetics. Our results demonstrate that the **Spiro-OMeTAD**-based SAMs have a great potential in producing low-cost iPSC devices, due to lower material usage, good long-term stability, and high performance.

## Introduction

Organic hole-selective materials are key for high-performance perovskite solar cells (PSCs).^1−3^ Specifically, the commercially available polymer **PTAA** and small molecule **Spiro-OMeTAD** are the most studied hole-selective contacts (HSCs) for developing high-efficiency devices, with a current 22.1% top efficiency for **PTAA** in a mesoporous structure and 25.8% for **Spiro-OMeTAD** in an n–i–p configuration.^4,5^ Additionally, **PTAA** also shows high efficiency for inverted (p–i–n) PSCs (iPSCs) and has advantages of long-term device operational stability, low-temperature fabrication, and suitability for perovskite-based tandem solar cells.^2,6^ However, its high cost (over 1000 euros per gram) and its hydrophobicity limit the low-cost commercialization of iPSCs. Alternatively, the relatively easily obtained and lower-cost **Spiro-OMeTAD** (∼200 euros per gram) is a good alternative choice as HSCs for inverted devices. In particular, **Spiro-OMeTAD** as HSCs possesses good reproductivity on device performance due to the well-defined molecular weight compared to polymers.^7,8^ Unfortunately, there have been few reported studies relating to **Spiro-OMeTAD** in iPSCs, and their efficiencies significantly lag behind those of **PTAA**-based devices, which drives our interest to focus on this topic.^9−11^

Pristine **Spiro-OMeTAD** suffers from low conductivity and hole mobility, as well as a large energy offset with perovskite, resulting in losses at the HSC/perovskite interface.^1,12^ Typically, **Spiro-OMeTAD** needs to be chemically doped with multiple dopants, such as LiTFSI, Co^III^TFSI, and 4-*tert-*butylpyridine (*t*BP), to improve its conductivity and hole mobility, as well as deepen the highest occupied molecular orbital (HOMO) for a better energy alignment at the interface.^13,14^ Nevertheless, the chemical dopants promote ion migration of the perovskite, induce interfacial nonradiative recombination, and deteriorate the long-term device stability.^15^ Self-assembled monolayers (SAMs) have been proven, by our group and others, to be highly promising HSCs in iPSCs.^16,17^ SAM molecules autonomously form the functional layer through chemical bonding to the substrate, offering the advantages of low material consumption and stable HSCs without dopants in iPSCs.^18^ The hole-selective SAMs were first applied to modify the ITO by using phosphonic acid and carboxylic acid as anchoring groups, replacing the traditional hole-transporting layer and showing high-performance in iPSCs.^16,19^ Later, various hole-selective SAM molecules were synthesized based on carbazole or phenothiazine units to improve the device performance, further demonstrating the promise of SAMs for iPSCs.^20,21^

In order to take advantage of **Spiro-OMeTAD** as a self-assembled monolayer, we synthesized a **Spiro-OMeTAD** derivative featuring a carboxylic acid unit as an anchoring group to form SAMs for HSCs in iPSCs (named **Spiro-Acid**). We demonstrate the high-performance of **Spiro-Acid**-based iPSCs with a power conversion efficiency (PCE) of 18.15%, which is comparable with that of **PTAA**-based iPSCs and the highest efficiency among **Spiro-OMeTAD**-based iPSCs, and they possess a remarkable stability under long-term illumination. This work highlights a new SAM molecule based on **Spiro-OMeTAD** as a HSC for fabricating cost-effective and high-performance iPSCs instead of classic thin films from spin-coating procedures that waste a large amount of the HSC material.

## Results and Discussion

**Figure 1:**
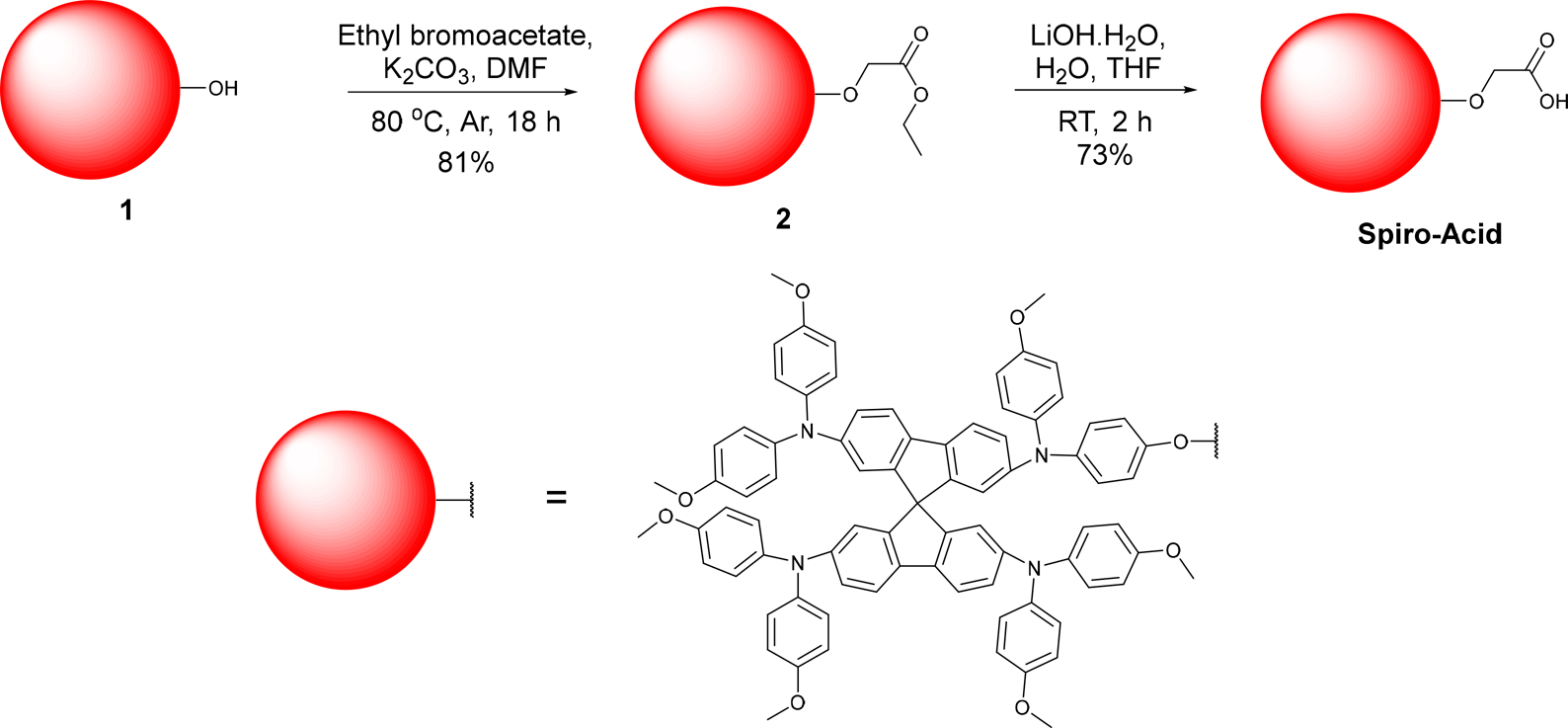
Synthesis of **Spiro**-**Acid**.

The synthesis of **Spiro-Acid** is outlined in Figure 1. Compound **1** was synthesized by monodemethylation of commercially available **Spiro-OMeTAD**, according to the procedure developed by Cooke et al.^22^ This was coupled, through a Williamson ether synthesis reaction, to commercially available ethyl bromoacetate, to obtain the ester **2**, which then underwent a saponification reaction with lithium hydroxide to give **Spiro-Acid** in good yield. One of the strengths of this synthetic approach is that inexpensive (unsublimed) **Spiro-OMeTAD** is used as the starting material, while the purification of **Spiro-Acid** and its precursor molecule **2** do not involve column chromatography (full details are provided in the Supporting Information). Furthermore, the synthesis could also be scaled up to multigram quantities. This easy-to-use methodology for the synthesis of molecules is extremely important in terms of commercialization.

The thermal properties of **Spiro-Acid** were studied by thermogravimetric analysis (TGA) and differential scanning calorimetry (DSC), as shown in Figure S1. TGA indicates that **Spiro-Acid** has a good stability up to 350 °C. However, above this temperature it starts to decompose. The DSC study was performed between 30 and 368 °C, with the upper limit being chosen by taking into consideration the TGA results. The lack of a crystallization temperature suggests that the material is highly amorphous. A glass transition is evident at 147 °C (*T*_g_), a value higher than that reported for **PTAA**, suggesting that **Spiro-Acid** should have a higher morphological stability than **PTAA** under continuous sunlight exposure.^23,24^

### Device Performance

**Figure 2:**
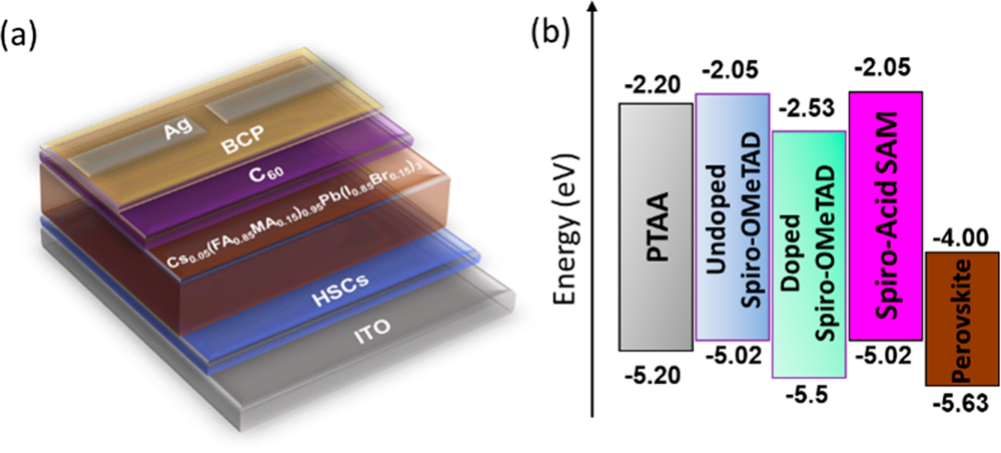
(a) Schematic of the p–i–n device architecture and (b) energy alignment of the different HSCs and perovskite used in this work.

To examine the potential effects of **Spiro-Acid** as a HSC on the device performance, the polymer **PTAA**, pristine **Spiro-OMeTAD** (**Spiro-undoped**) and chemically doped **Spiro-OMeTAD** (**Spiro-doped**) were also used as reference HSCs in iPSCs. Therefore, four different HSCs were used in the following device structure: ITO/HSCs/Cs_0.05_(FA_0.85_MA_0.15_)_0.95_Pb(I_0.85_Br_0.15_)_3_/C_60_/BCP/Ag, as schematically shown in Figure 2a. In addition, the energy levels of the different HSCs are illustrated in Figure 2b. The energy levels of **PTAA**, **Spiro-undoped**, and **Spiro-doped** have been reported in the literature,^25^ while the energy levels of **Spiro-Acid** were estimated from square wave voltammetry (Figure S2), which shows an oxidation potential similar to that of **Spiro-OMeTAD**. Since the conductivity and hole mobility can be affected by the exposure conditions, we optimized the devices by depositing all Spiro-based HSCs under either N_2_ or air (see Figure S4 and Table S1).^26^**Spiro-undoped** prepared in air, **Spiro-doped** prepared in N_2_, and **Spiro-Acid** prepared in N_2_ show better device performance compared to **Spiro-undoped** prepared in N_2_, **Spiro-doped** prepared in air, and **Spiro-Acid** prepared in air, respectively. The reason only the undoped **Spiro-OMeTAD** behaves better when it is prepared in air has been previously addressed and is due to the increased concentration of oxidized **Spiro-OMeTAD**, which enhanced the conductivity for **Spiro-undoped** and avoided absorption of moisture that could affect perovskite crystal growth.^27,28^ On the other hand, HSC-free devices were fabricated to further confirm that the enhanced device performance comes from the introduction of Spiro-based HSCs. We also investigated the devices with **Spiro-Acid**, formed by either dipping or spin-coating processes (Figure S5 and Table S2). The better performance from the dipping process compared with the spin-coating process arises from the significant enhancement of the fill factor (FF), mainly resulting from the formation of an ultrathin film by the dipping method and the reduced resistance for interfacial charge transfer. This further indicates that the **Spiro-Acid**-dipped film can be suitable for large-area applications. Therefore, the following comparison and analysis will be based on **Spiro-undoped** prepared in air, **Spiro-doped** prepared in N_2_, and **Spiro-Acid** prepared in N_2_ through the dipping process, as well as **PTAA** by spin-coating as the reference.

**Figure 3:**
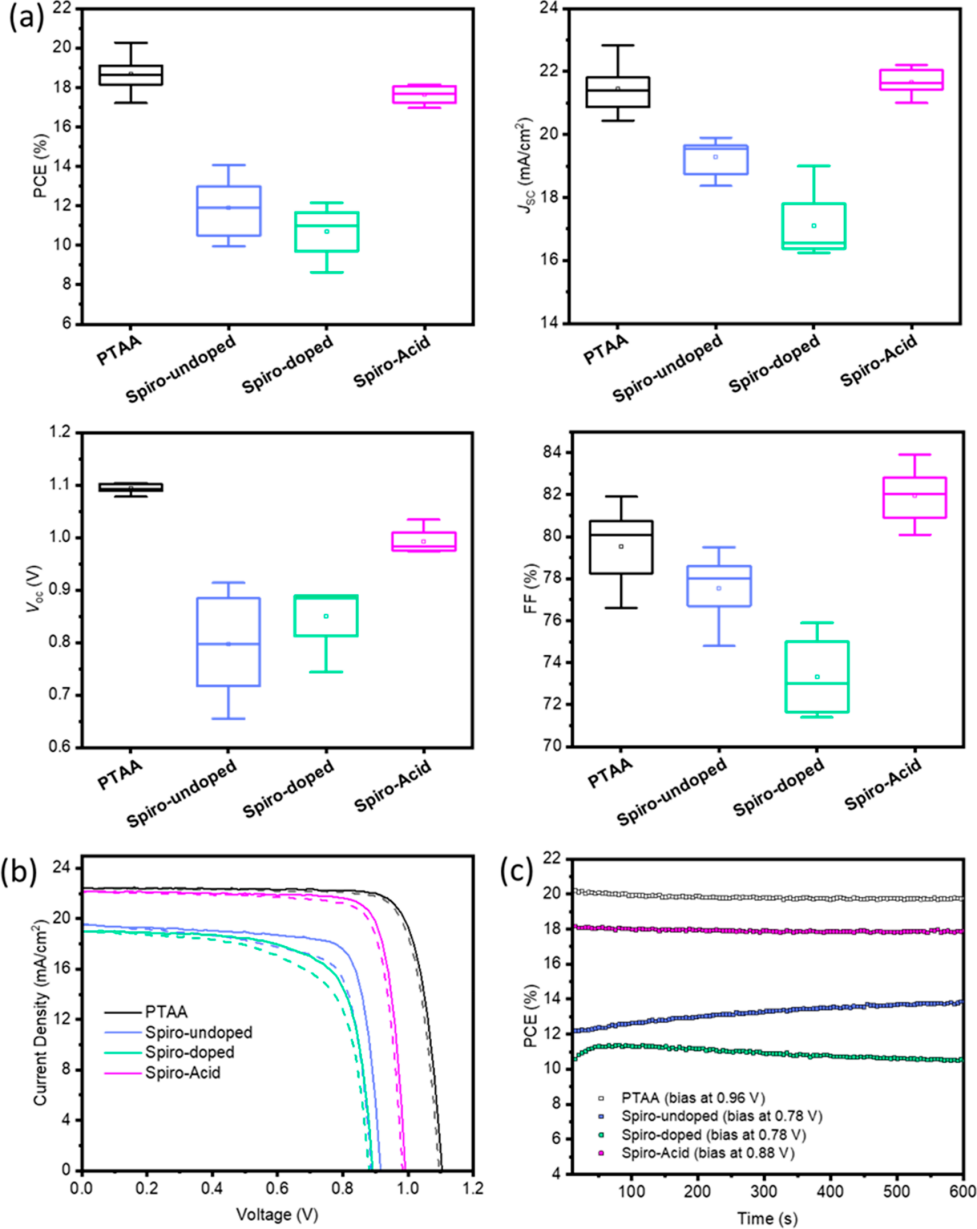
(a) Device performance statistics with the different HSCs, (b) *J*–*V* curves from the champion cells with the corresponding HSC-based iPSCs, and (c) quasi-steady-state efficiency by MPP tracking with the different HSC-based devices.

In order for us to reliably compare the device performance based on different HSCs, neither bulk nor interfacial defect passivation were used in this work. Figure 3a and Table 1 show the statistical distribution of the device parameters. Clearly, the **Spiro-Acid**-based devices show significantly higher performance compared with **Spiro-undoped**- and **Spiro-doped**-based cells and efficiencies comparable with those of **PTAA** devices. We could attribute the lower performance of **Spiro-undoped**- and **Spiro-doped**-based devices to the low conductivity of pristine **Spiro-OMeTAD** and also to the large dopant-induced interfacial nonradiative recombination in **Spiro-doped**-based cells, respectively.^11,29^ We can also observe that the different PCEs between **Spiro-Acid** SAM- and **PTAA**-based devices are mainly caused by the lower *V*_OC_ (approximately 100 mV), due to the larger energetic offset between the HOMO of the SAM and the valence band (VB) of the perovskite, as well as the carrier recombination kinetics in SAM-based devices, which will be discussed in detail later. Nonetheless, we estimated the energy loss at the interface between perovskite and the HSC based on the equation Δ*V*_loss_ = (*E*_CB,PVK_ – *E*_HOMO,HSC_)/*q* – *V*_OC_. The energy losses are 0.097 V for **PTAA**, 0.106 V for **Spiro-undoped**, 0.610 V for **Spiro-doped**, and 0.030 V for **Spiro-Acid**. The **Spiro-Acid**-based PSC has the lowest energy loss at the interface, indicating that its *V*_OC_ is approaching the theoretical voltage based on the energy alignment. Furthermore, the SAM **Spiro-Acid**-based devices demonstrated impressive FFs, with values of over 82%, indicating the low resistance for interfacial charge transfer.

**Table 1:** Photovoltaic Parameters of the Champion Cells and the Statistics of an Average of 10 Devices Based on Different HSCs

HSC	sweep	*J*_SC_ (mA/cm^2^)	*V*_OC_ (V)	FF (%)	PCE (%)
**PTAA**	reverse	22.44 (21.45 ± 0.73)	1.103 (1.094 ± 0.008)	81.9 (79.5 ± 1.7)	20.27 (18.67 ± 0.89)
	forward	22.44	1.096	81.3	20.00
Spiro-undoped	reverse	19.53 (19.28 ± 0.55)	0.914 (0.797 ± 0.099)	78.8 (77.5 ± 1.5)	14.07 (11.92 ± 1.57)
	forward	19.55	0.890	72.1	12.54
**Spiro-doped**	reverse	19.00 (17.10 ± 1.28)	0.890 (0.851 ± 0.071)	71.9 (73.3 ± 2.1)	12.15 (10.69 ± 1.48)
	forward	19.01	0.880	66.2	11.08
**Spiro-Acid**	reverse	22.20 (21.66 ± 0.43)	0.990 (0.993 ± 0.024)	82.6 (82.0 ± 1.4)	18.15 (17.63 ± 0.46)
	forward	22.15	0.984	81.2	17.70

Figure 3b shows the *J*–*V* curves of the best devices with **PTAA**, **Spiro-undoped, Spiro-doped**, and **Spiro-Acid** as the HSCs. The **PTAA** champion cell performance, with 20.27% PCE, is comparable to others reported in the literature, indicating that our device fabrication process is reliable for the comparison.^30^ Significantly, the best PCE of the **Spiro-Acid** device is 18.15% with negligible hysteresis, which is higher than those obtained for **Spiro-OMeTAD**-based iPSCs reported in the literature (Table S3). We summarized the evolution of efficiency in iPSCs based on **PTAA**, SAMs, and **Spiro-OMeTAD** (Figure S6), indicating the promising nature of the SAM technology and its application to **Spiro-OMeTAD** to further improve the efficiency of iPSCs. Furthermore, the integrated *J*_SC_ value of the best SAM device obtained by using the external quantum efficiency (EQE) technique is shown in Figure S7. The efficiency of the **Spiro-Acid** device surpassed those of **Spiro-undoped** (14.07%) and **Spiro-doped** (12.15%), approaching that of **PTAA**-based cells with a similar *J*_SC_ and FF, but lower *V*_OC_ (0.990 V versus 1.103 V). In addition, quasi-steady-state efficiency by maximum power point (MPP) tracking shows a stable output of 18.07%, close to the *J*–*V* value from the **Spiro-Acid** device, while **PTAA** device decreases slowly and **Spiro-undoped** and **Spiro-doped** devices are unstable within 600 s tracking time, as shown in Figure 3c. All photovoltaic parameters of the different HSC-based devices are provided in Table 1. These findings indicate the promise for **Spiro-Acid** as a HSC for the fabrication of high-performance iPSCs.

### Morphology Characterization

**Figure 4:**
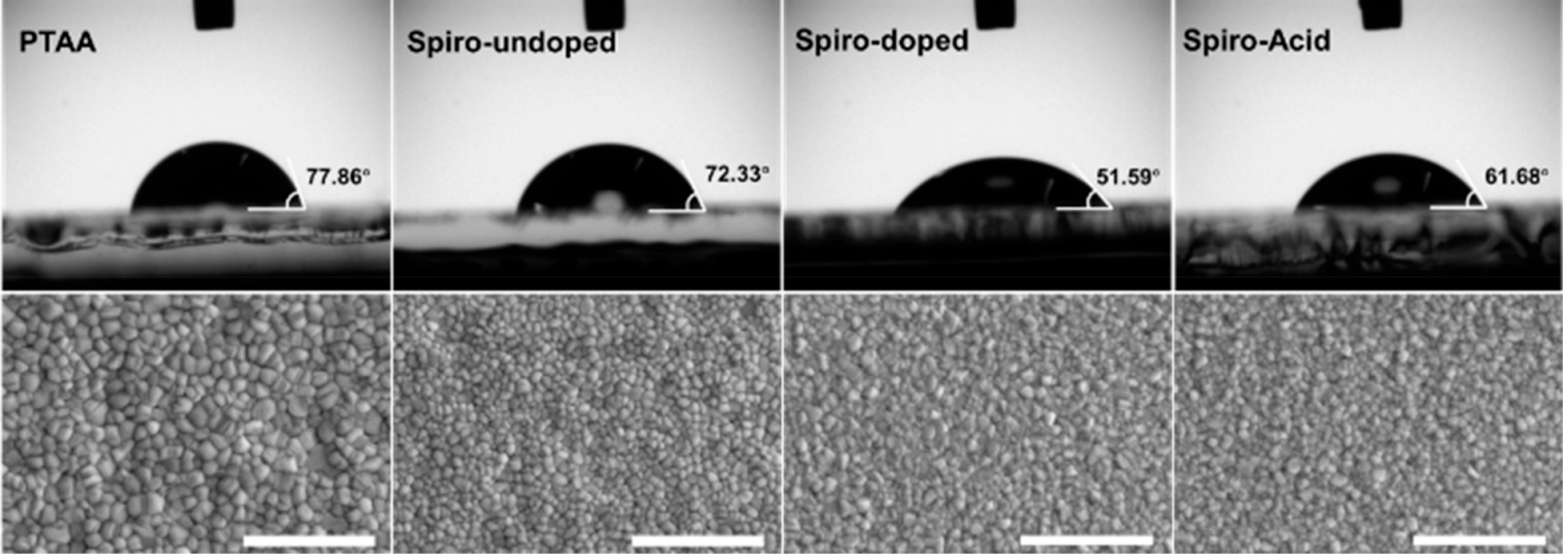
Contact angle of different HSC surfaces and the FESEM top images of perovskite films deposited on the corresponding HSCs. All scale bars are 2 μm.

We employed contact angle measurements and field emission scanning electron microscopy (FESEM) to determine the surface wettability of different HSCs and their effect on the morphology of perovskite crystals, as shown in Figure 4. The **PTAA** layer shows higher hydrophobicity than the other HSCs. Conversely, **Spiro-Acid** shows a lower contact angle of 61.68° compared to **PTAA**, indicating the easy deposition of the perovskite solution onto the SAM surface. Additionally, the contact angle of the SAM is higher than that of bare ITO (55.38°) after UV–ozone treatment (Figure S8) but lower than that of **Spiro-undoped** (72.33°). The hydrophobic surface of **PTAA** leads to a large grain size of the perovskite (average grain size of 230 nm), while **Spiro-Acid** results in small grains with a dense perovskite film (average grain size of 165 nm), as shown in FESEM images in Figure 4 and grain size distributions in Figure S9. In general, a large grain size with reduced grain boundaries can decrease the defect density at the perovskite film, which will facilitate the charge transfer. In addition, we also observed that there are no PbI_2_ plates present in the **Spiro-Acid** film as compared with the **PTAA** film. It is reported that perovskite with excess/unreacted PbI_2_ can lower the long-term stability of devices under continuous illumination, due to the photodecomposition of unreacted PbI_2_ into metallic Pb and I_2_ under light irradiation, which act as nonradiative recombination centers for quenching carriers and then gradually decrease the PCE of cells.^31^ Further details of the illumination stability will be discussed in the following section.

### Photoluminescence

**Figure 5:**
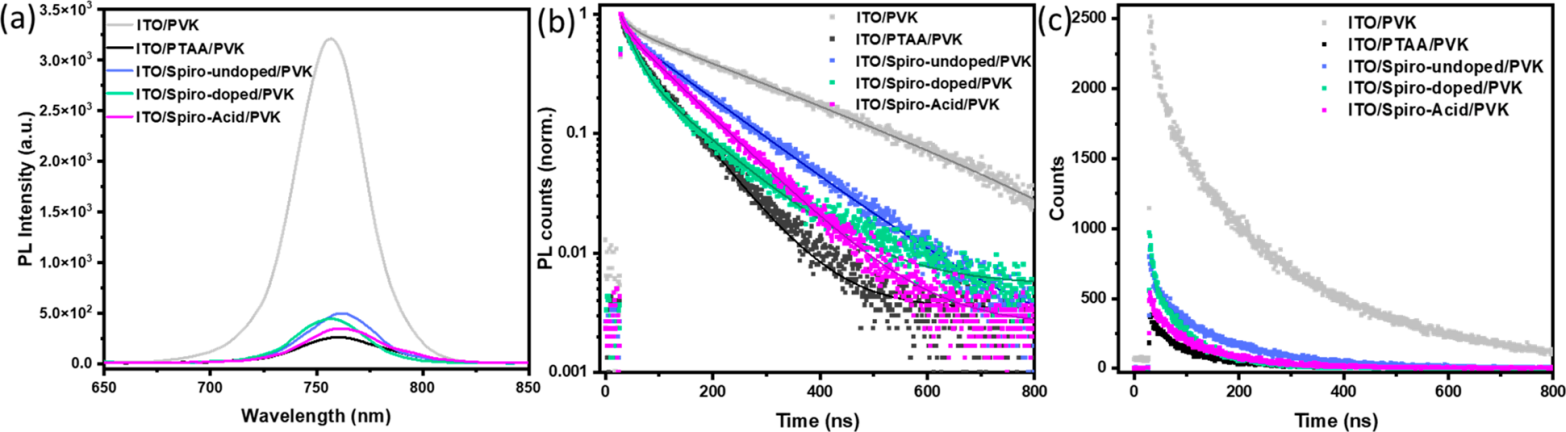
(a) Steady-state photoluminescence spectra and (b) normalized time-resolved photoluminescence decays with a fixed 3000 acquisition counts and (c) with a fixed 300 s acquisition time, after laser excitation at 635 nm for perovskites deposited either on ITO or on different ITO/HSC substrates. All samples were illuminated from the glass side. PVK denotes the perovskite.

Steady-state photoluminescence (PL) and time-resolved photoluminescence (TRPL) were used to probe the charge transfer dynamics at the perovskite/HSC interfaces. Compared to the perovskite film on bare ITO, perovskite films on all HSCs show a significant decrease in PL intensity (Figure 5a), owing to the luminescence quenching at the perovskite/HSC interfaces (charge transfer to HSCs). We further monitored the charge dynamics by using TRPL with a fixed acquisition count, as shown in Figure 5b. The decay curves were fitted with the biexponential decay function *y* = *A*_1_ exp(−*t*/τ_1_) + *A*_2_ exp(−*t*/τ_2_), where τ_1_ and τ_2_ represent fast and slow decay time components associated with trap-assisted charge recombination of the perovskite and free-carrier transfer at the interfaces and radiative charge recombination, respectively.^5^ Decay fitting parameters are given in Table S4. The lifetimes τ_1_ present the trend **PTAA** (9.9 ns) < **Spiro-undoped** (12.0 ns) ≈ **Spiro-Acid** (12.3 ns) < ITO (18.7 ns) < **Spiro-doped** (22.4 ns), while the lifetimes τ_2_ have the trend **PTAA** (75.8 ns) < **Spiro-Acid** (99.3 ns) < **Spiro-doped** (111.4 ns) < **Spiro-undoped** (131.8 ns) < ITO (251.6 ns). It should be noted that the **Spiro-doped**-based sample presents different decay behavior compared to that of other HSCs, probably owing to the dopants acting as nonradiative recombination centers for fast-quenching carriers.^32^ The shortest lifetimes (both τ_1_ and τ_2_) are from **PTAA**, for which the PL intensity is also the most reduced, implying that the **PTAA**/perovskite interface can reduce the nonradiative interfacial recombination, as well as allow a fast charge transfer from the perovskite to **PTAA**. In contrast, the **Spiro-Acid**-based sample shows lifetimes slightly slower than that of **PTAA**, which is further confirmed from the PL decays with a fixed acquisition time (538 counts from **Spiro-Acid** and 378 counts from **PTAA** within 300 s acquisition time), as shown in Figure 5c and Table S4. Therefore, the relatively adverse charge recombination at the **Spiro-Acid**/perovskite interface in comparison to the **PTAA**/perovskite interface is the possible reason for the lower *V*_OC_ in **Spiro-Acid** devices compared to that of **PTAA**.

### Photophysics

These devices were also characterized by using advanced transient techniques such as charge extraction (CE), transient photovoltage (TPV), and transient photocurrent (TPC) in order to analyze the carrier recombination when different HSCs were used. These techniques have been previously used by our group and others, to analyze the recombination kinetics in different emerging photovoltaic technologies, as dye-sensitized solar cells (DSSCs), quantum dots, organic photovoltaics, and most recently perovskite solar cells.^33−36^ Basically, CE and TPC (via differential capacitance, DC) techniques are used to estimate the charge density at different light biases and the TPV technique is used to study carrier recombination, all under close *operando* conditions.^37^ The CE technique can be used as long as the CE decay is faster than the TPV decay when comparing the same light intensity. Therefore, Figure S10 compares all the different devices under 1 Sun conditions in order to elucidate if the CE method can be used instead of DC method—a combination of TPC/TPV. As can be seen, all of the monoexponential CE decays were faster than the TPV decays, indicating that carrier collection is faster than carrier recombination. To accurately analyze the charge density of the devices, we used both CE and DC (see an example in Figure S11).

**Figure 6:**
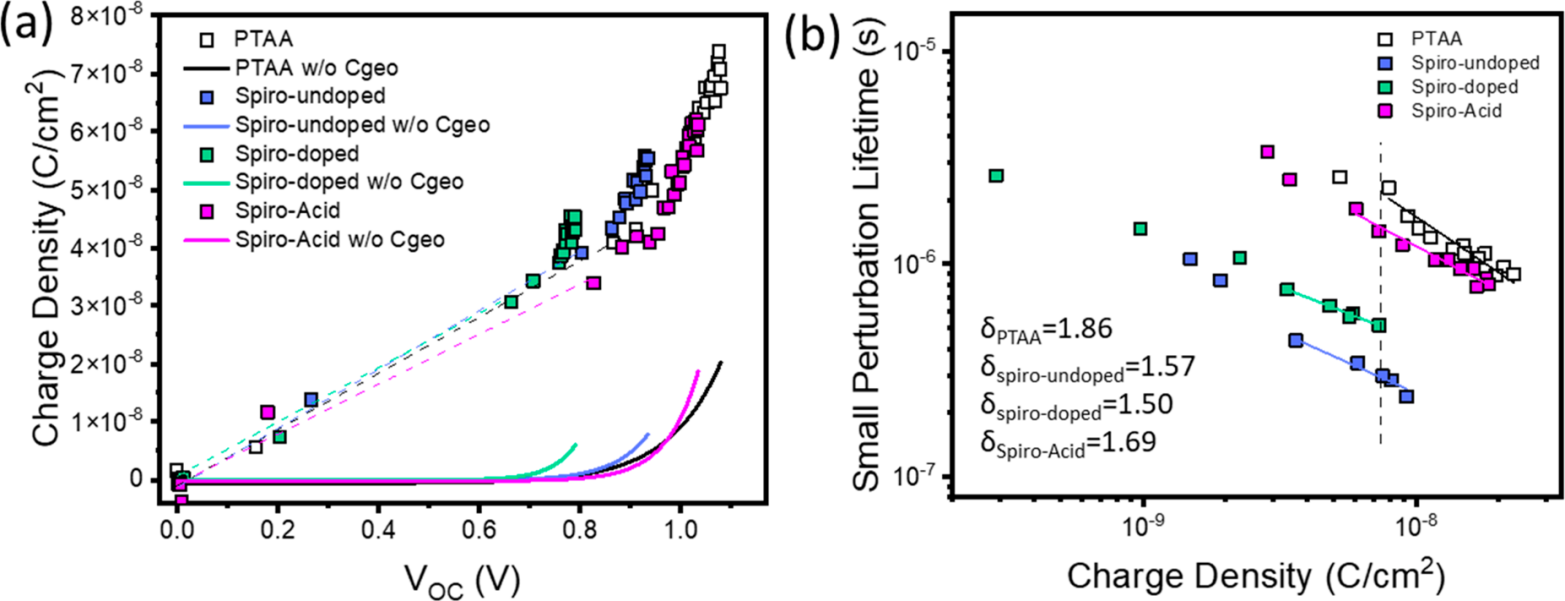
(a) Charge density from CE as a function of voltage for the different HSCs used in this work. The filled symbols represent the geometrical (*C*_geo_) and chemical capacitance. The solid lines at the bottom correspond to the charge density after subtracting *C*_geo_. (b) Small perturbation lifetime from TPV as a function of charge density in the devices after subtracting *C*_geo_.

Figure 6a shows the results of CE for the p–i–n perovskite solar cells when using **PTAA**, **Spiro-undoped**, **Spiro-doped**, and **Spiro-Acid** as HSCs. Two regimes are observed. First, there is a constant part which is directly related to the geometrical capacitance (*C*_geo_) or, in other words, to charges stored at the electrodes. Devices containing **Spiro-Acid** are linked to a slightly smaller capacitance. This can be attributed to the lower permittivity of the SAM in comparison to the other HSCs, probably due to the fact of having a single layer in the solar cell structure. Second, there is an exponential part, which has been linked to the chemical capacitance or, in other words, to the accumulated charges in the perovskite/selective-contacts interface. Therefore, after the subtraction of *C*_geo_, we can obtain a direct estimation of the charge density in the bulk of the perovskite. We can clearly observe that, as expected, the higher the photocurrent obtained from the *J*–*V* characteristics, the higher the charge density.

From Figure S12, we observed that faster recombination lifetimes are obtained under 1 Sun conditions for **Spiro-doped** and **Spiro-undoped.** However, since similar voltage values lead to different charge densities, it is better to compare the recombination kinetics as a function of charge density (see Figure 6b). When comparing the recombination lifetimes at the same charge density (indicated by a dashed line), we observed that both **Spiro-doped** and **Spiro-undoped** have faster recombination compared to **Spiro-Acid** and **PTAA** HSCs. Hence, a possible relationship with the perovskite grain size—or grain boundary density—needs to be carefully analyzed, since as reported by other authors, the grain boundaries in the perovskite layer can act as trap-assisted recombination channels and be detrimental for the charge-carrier collection but also its presence in polycrystalline solar cells has also shown positive effects.^38,39^ Finally, Figure 6b can be fitted to a power law dependence following the equation , where λ is the slope and can be correlated with the recombination order via δ = λ + 1.^40^ In general, all the recombination orders obtained are alike and are ruled mainly by a second order (δ = 2) corresponding to bimolecular recombination pathways, which have been already observed in perovskite solar cells.^41^

### Long-Term Illumination Stability

**Figure 7:**
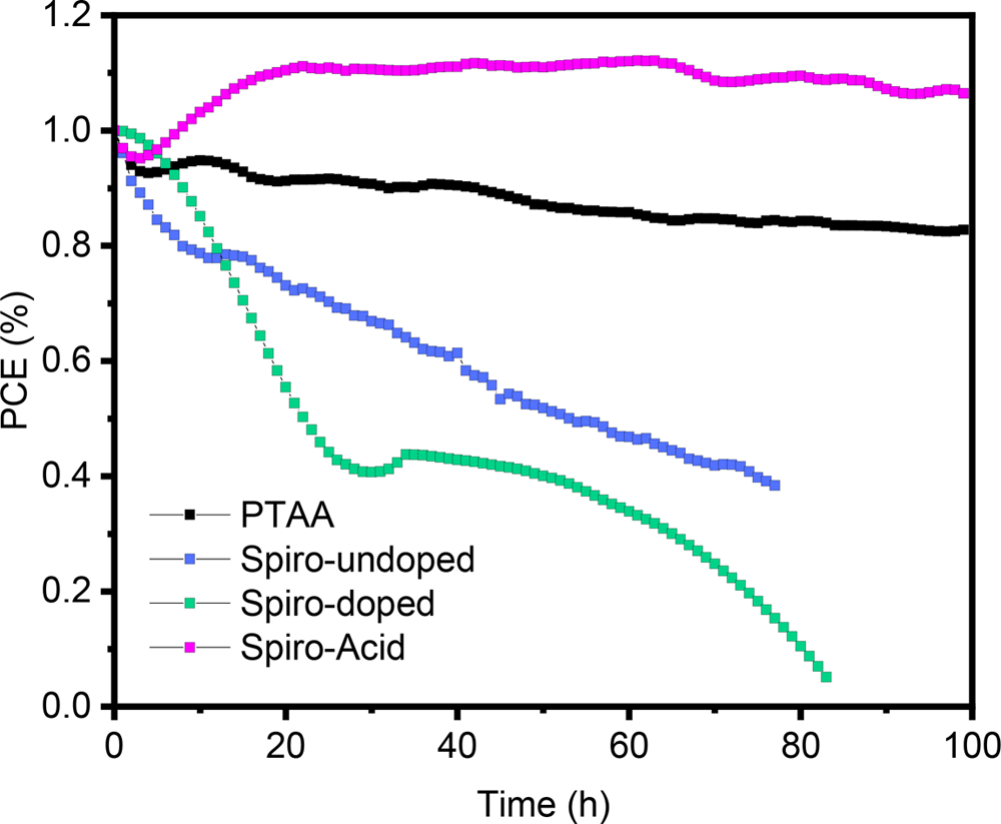
Long-term continuous illumination of PSCs based on different HSCs.

The long-term illumination stability was determined for the different devices at a simulated 1 Sun AM 1.5G illumination, as shown in Figure 7 and Figure S13. The samples were stored in a homemade N_2_-filled holder and tested at room temperature. The **Spiro-doped** HSC device rapidly decreased to 40% of the initial PCE after 30 h and nearly to 10% after 80 h, due to the presence of dopants that accelerates the degradation under continuous illumination, as shown in the optical image of the device after the test (Figure S13). Additionally, the **Spiro-undoped** HSC device is also unstable under illumination, only maintaining 40% of the initial PCE after 80 h. In contrast, **PTAA** and **Spiro-Acid** HSC devices show better stability than the other two HSCs, but the **PTAA** cell gradually decreases to 82% of the initial PCE at around 100 h. Significantly, the **Spiro-Acid** SAM cell shows ultrahigh stability with a gradual increase and then a slow decrease in PCE within 100 h and nearly unchanged device appearance, demonstrating its superior long-term illumination stability. Previous work has demonstrated that **PTAA** devices are unstable under UV light due to the UV-induced degradation of **PTAA** by breaking the aromatic functional groups.^42^ However, the UV absorption is negligible in the SAM HSC due to the ultrathin layer produced by the dipping process. Furthermore, the enhanced illumination stability of the SAM device is also due to the removal of PbI_2_ from the perovskite, which can be photodecomposed by forming nonradiative recombination centers and reducing the efficiency.

## Conclusions

In this work, we have shown that **Spiro-Acid** can be used as an effective hole transport material without dopants in p–i–n perovskite solar cells. To the best of our knowledge it is the first time that **Spiro-OMeTAD** has been used as a self-assembled molecule in optoelectronic devices. The SAM-based device shows a similar efficiency with an outstanding fill factor in comparison to **PTAA**-based devices and, moreover, excellent long-term illumination stability. Transient optoelectronic measurements were applied to films and complete devices in this work to understand the interfacial carrier recombination kinetics. Such carrier losses are comparable to standard devices using **PTAA**. Nonetheless, the energy loss in **Spiro-Acid**-based perovskite solar cells is the smallest in all comparisons. This work paves the way for future modifications of **Spiro-OMeTAD** as a SAM and the fabrication of efficient and stable p–i–n perovskite solar cells without the use of chemical dopants at the hole transport layer.

## Experimental Section

### Device Fabrication

Prepatterned ITO glass substrates (1.5 cm × 1.5 cm, 15 Ω sq^–1^) were sequentially cleaned with ethanol and IPA for 15 min, respectively. The ITO substrates were dried with N_2_ gas and treated with UV–ozone before use for 20 min.

#### **PTAA**

A 2 mg/mL portion of **PTAA** (Sigma-Aldrich) in anhydrous toluene (Sigma-Aldrich) was prepared and spin-coated at 6000 rpm for 30 s and then annealed at 100 °C for 10 min in an N_2_ glovebox.

#### Undoped **Spiro-OMeTAD**

**Spiro-OMeTAD** (1-Material) was dissolved in anhydrous toluene with a concentration of 1 mM and spin-coated at 3000 rpm for 30 s in N_2_ or air, and then annealed at 100 °C for 10 min in N_2_ or air.

#### Doped **Spiro-OMeTAD**

A 1 mM solution of **Spiro-OMeTAD** in anhydrous toluene was prepared and doped by adding 0.4 μL of *t*BP, 0.24 μL of LiTFSI (520 mg/mL in acetonitrile), and 0.14 μL of Co^III^TFSI (300 mg/mL in acetonitrile), and the spin-coating steps were the same as those for the undoped **Spiro-OMeTAD**.

#### **Spiro-Acid**

For the dip-coating process, ITO substrates were immersed into 0.1 mM **Spiro-Acid** in anhydrous toluene solution at 50 °C for 6–10 h in a sealed container. Then the dipped substrates were dynamically washed with toluene two to three times at 3000 rpm for 30 s in N_2_ or air and annealed at 100 °C for 10 min in N_2_ or air. For the spin-coating process, **Spiro-Acid** was dissolved in anhydrous toluene with a concentration of 1 mM and then spin-coated at 3000 rpm for 30 s and annealed at 100 °C for 10 min in N_2_.

The triple-cation perovskite layer was deposited in an N_2_ glovebox. Briefly, a perovskite precursor with excess PbI_2_ composed of FAI (1.1 M), PbI_2_ (1.15 M), MABr (0.2 M), and PbBr_2_ (0.2 M) was dissolved in anhydrous DMF DMSO (4/1 v/v). Then 42 μL of a CsI stock solution (1.5 M in DMSO) was added to the mixed perovskite solution. The perovskite solution was spin-coated onto **PTAA** (prewetted by DMF) or Spiro-based HSCs in a two-step procedure at 2000 rpm for 10 s and 4000 pm for 25 s. A 110 μL portion of chlorobenzene was dropped on the spinning substrate at the last 12 s. Then, the samples were annealed at 100 °C for 60 min.

After perovskite deposition, samples were transferred into a thermal evaporator for C_60_ (23 nm) and BCP (9 nm) deposition. Subsequently, a 100 nm Ag layer was evaporated at low pressure (<10^–6^ bar) defining an area of 0.09 cm^2^.

### Device Characterization

The *J*–*V* curves were recorded using a solar simulator (ABET 11000) and a source meter (Keithley 2400). The curves were registered under 1 Sun conditions (100 mW/cm^2^, AM 1.5G) calibrated with a Si-reference cell. The scan rate employed was 80 mV/s. The active area of the devices was 0.09 cm^2^. The EQE was measured by quantum efficiency measurement systems from Lasing, S.A. (IPCE-DC, LS1109-232) and a Newport 2936-R power-meter unit. Long-term illumination was performed in a homemade LED white light system. All devices were sealed in a holder under room temperature in an N_2_ atmosphere. The photovoltage parameters were automatically recorded every 60 min by the software. Field emission scanning electron microscopy (FESEM) was used with an FEI Quanta 600 microscope to obtain the surface morphology of perovskites. Contact angle measurements were performed with an optical tensiometer (Attention Theta Flex, Biolin Scientific, Sweden) using a sessile drop analysis.

Steady-state photoluminescence (PL) and time-resolved photoluminescence (TRPL) spectra were obtained on an Edinburgh Instruments LifeSpec-II apparatus with excitation by a 635 nm laser. All perovskite films were protected with PMMA to do measurements under ambient conditions.

Photoinduced charge extraction (CE), transient photovoltage (TPV), and transient photocurrent (TPC) measurements were carried out using a white LED controlled by a programmable power supply and a control box that switched from open- to short-circuit states. All the signals were recorded using an Yokogawa DLM2052 oscilloscope registering drops in voltage. Light perturbation pulses for TPV and TPC were provided by a nanosecond PTI GL-3300 nitrogen laser and using a 580 nm laser pulse wavelength.

## Supporting Information Available

Synthetic details, ^1^H NMR, ^13^C NMR, cyclic voltammetry, and device performance (PDF)
