# Supplementary material for: Self-Assembled Molecules for Hole-Selective Electrodes in Highly Stable and Efficient Inverted Perovskite Solar Cells with Ultralow Energy Loss
Source: ACS Appl Energy Mater. 2023 Jan 13;6(3):1239–47. doi: 10.1021/acsaem.2c02880 (PMC9930087; doi:10.1021/acsaem.2c02880)
Supplement: Supplementary file 1 — ae2c02880_si_001.pdf [file ae2c02880_si_001.pdf]

Supporting Information

Self-assembled molecules for hole-selective electrodes in highly stable and efficient inverted perovskite solar cells with ultra-low energy loss.

Wenhui Li[#1], Michele Cariello[#2], Maria Méndez[1], Graeme Cooke[*2], and Emilio Palomares[*1,3]

[1] Institute of Chemical Research of Catalonia (ICIQ-BIST), Avda. Països Catalans, 16, 43007 Tarragona, Spain

[2] School of Chemistry, University of Glasgow, Glasgow, G12 8QQ, UK.

[3] Catalan Institution for Research and Advanced Studies (ICREA), 08010 Barcelona, Spain

# these authors contribute equally

corresponding authors: Graeme.Cooke@glasgow.ac.uk, epalomares@iciq.es

1. Synthesis

All reagents and starting materials were obtained from commercial sources and used as received. Compound **1** was synthesised according to the previously reported procedure.[1] Merck silica gel (60 Å) covered aluminium plates F254 were used for thin layer chromatography. ^{1}H and ^{13}C NMR spectra were obtained with a Bruker AVIII 400 MHz spectrometer with chemical shift values in ppm relative to benzene: 7.16 (^{1}H) and 128.06 (^{13}C). Coupling constants, J, are reported in Hz and the following abbreviations are used to label the multiplicities: s, singlet; d, doublet; t, triplet and m, multiplet. Mass spectrometry data (ESI) were obtained at the University of Glasgow using either a Bruker MicroTOF-Q or an Agilent 6200 Series TOF/6500 series Q-TOF 10.1.

Electrochemistry was performed using a CH Instrument Electrochemical Workstation (CHI 440a), Austin, TX, USA. Ferrocene was used as an external standard, with the ferrocenium/ferrocene (Fc^+/Fc) redox couple adjusted to 0.0 V. The solutions were prepared using anhydrous dichloromethane containing 5 × 10^{-4} M concentration of sample and electrochemical grade tetrabutylammonium hexafluorophosphate (0.1 M) as the supporting electrolyte. The solutions were purged with N_2 gas for 3 minutes prior to recording the electrochemical data.

Synthesis of compound 2

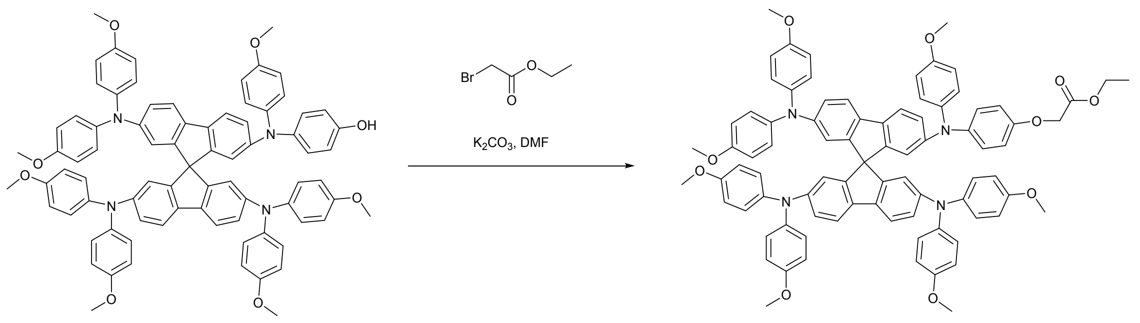

1 2

Under Ar, compound **1** (0.500 g, 0.413 mmol) and potassium carbonate (0.285 g, 2.06 mmol) were dissolved in anhydrous DMF (50 mL). The mixture was stirred at 80 °C for 30 minutes, then ethyl bromoacetate (0.46 mL, 4.13 mmol) was added and the resulting mixture was left to stir at 80 °C for 18 hours. The solvent was then evaporated, and the residue was dissolved in ethyl acetate (100 mL). This was washed with an aqueous solution of saturated ammonium chloride (80 mL), water (80 mL) and brine (80 mL), successively. The organic extract was dried over magnesium sulfate, filtered and concentrated under vacuum. The resulting residue was dissolved a minimum amount of ethyl acetate and added drop-wise to methanol (150 mL) while stirring. The precipitate was collected by vacuum filtration, washed with more methanol, and dried under vacuum, affording compound **2** (0.434 g, 81%) as an off-white solid. mp > 300 °C. R_f = 0.7 (hexane:ethyl acetate = 65:35). ^{1}H-NMR (C_6D_6, 400 MHz) δ_H 7.14 – 6.93 (28H, m), 6.78 – 6.70 (16H, m), 4.15 (2H, s), 3.88 (2H, q, *J* = 7.1 Hz), 3.28 – 3.24 (21H, m), 0.86 (3H, t, *J* = 7.1 Hz). ^{13}C-NMR (C_6D_6, 100 MHz) δ_C 168.56, 156.08, 155.96, 150.85, 148.23, 142.01, 135.88, 128.69, 126.10, 125.85, 125.11, 122.98, 120.67, 118.33, 115.81, 114.95, 66.49, 65.88, 60.79, 54.95, 54.93, 14.10. HRMS (ESI) m/z: $[M]^+$ Calcd for $C_{84}H_{72}N_4O_{10}$ 1296.5243; found 1296.5243.

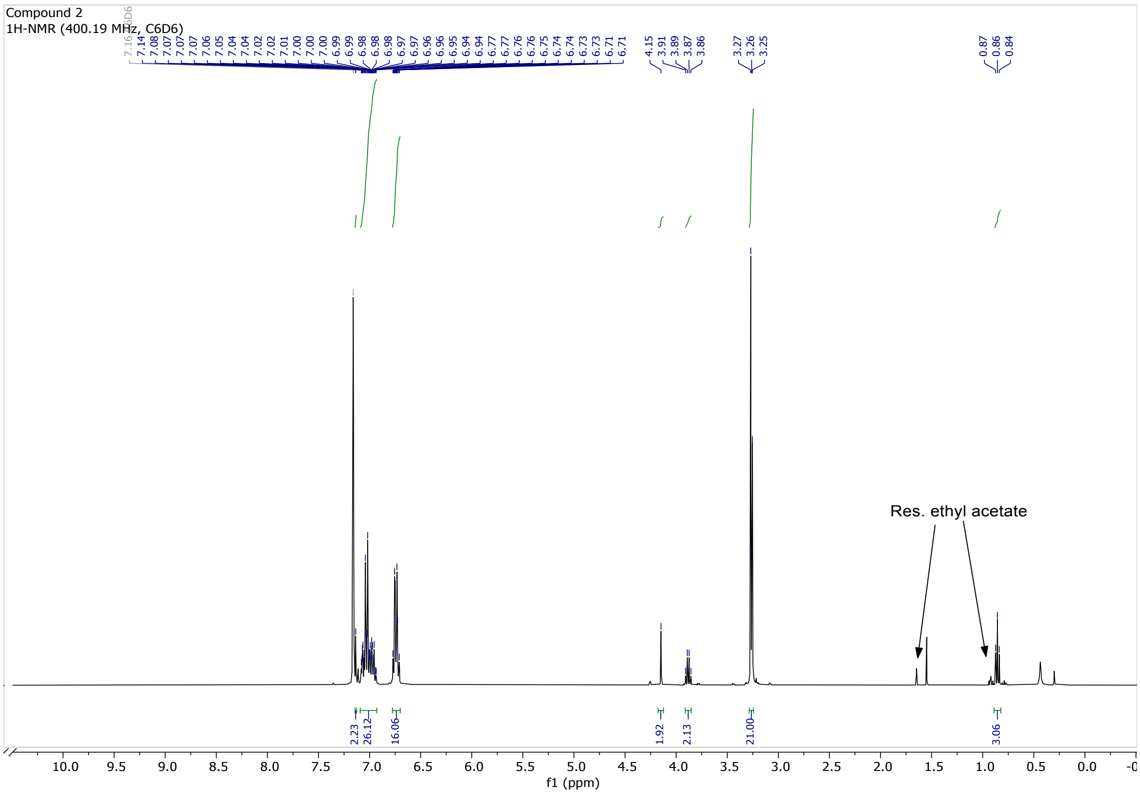

^{1}H NMR of compound

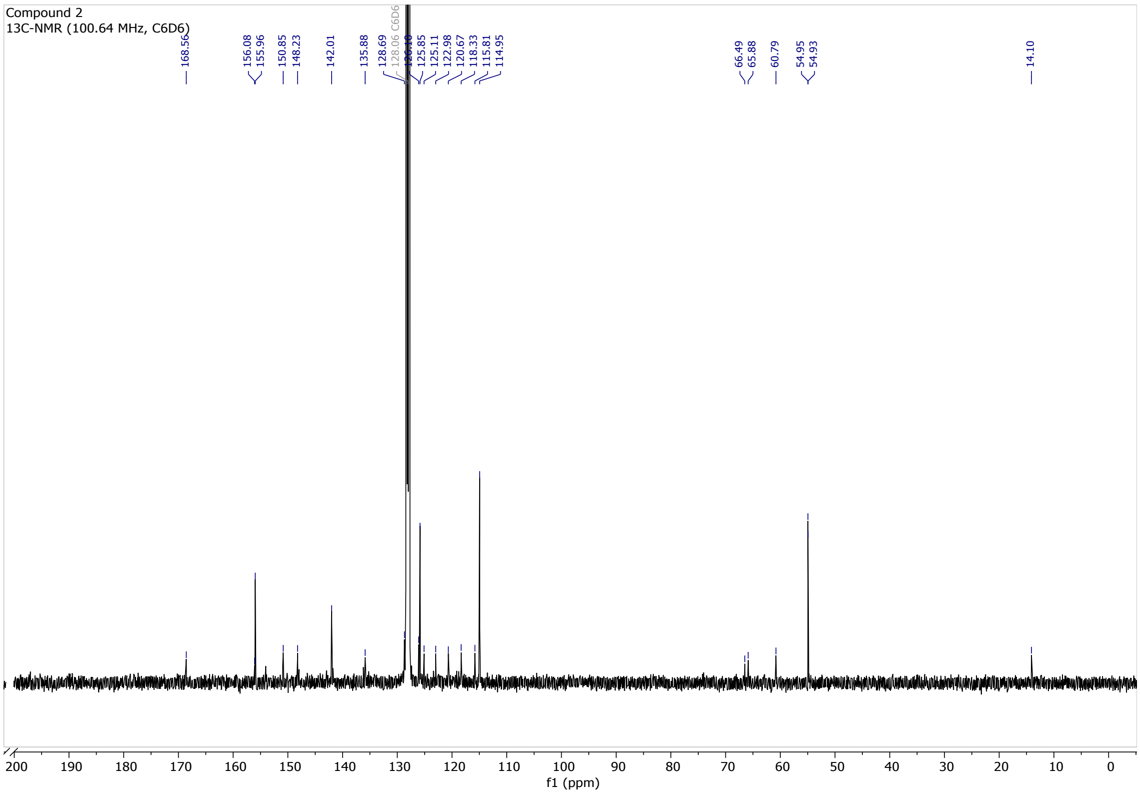

^{13}C NMR of compound **2**

Mass Spectrum SmartFormula Report

Analysis Info

Analysis Name D:\Data\Mass Spectrometry Service\77709-000002.d
Method LM MS 50 to 1500.m
Sample Name Cariello-MC-843
Comment

Acquisition Date 6/2/2021 8:45:25 AM
Operator user
Instrument / Ser# micrOTOF-Q 74

Acquisition Parameter

Source Type ESI Ion Polarity Positive
Scan Begin 50 m/z
Scan End 1500 m/z

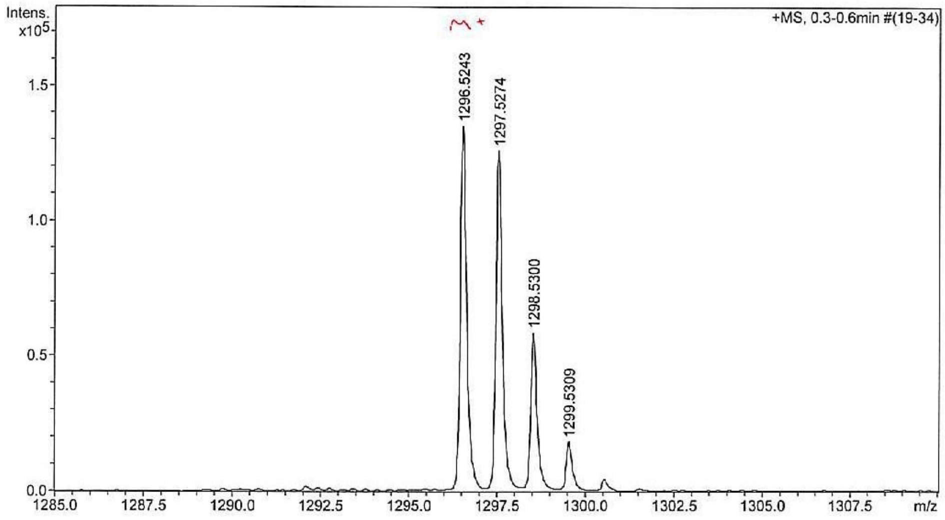

Formula	z	m/z	Meas. m/z	err [ppm]	err [mDa]
	1+		1296.5243		
C 84 H 72 N 4 O 10		1296.5243		0.0	0.0

*Synthesis of **Spiro-Acid***

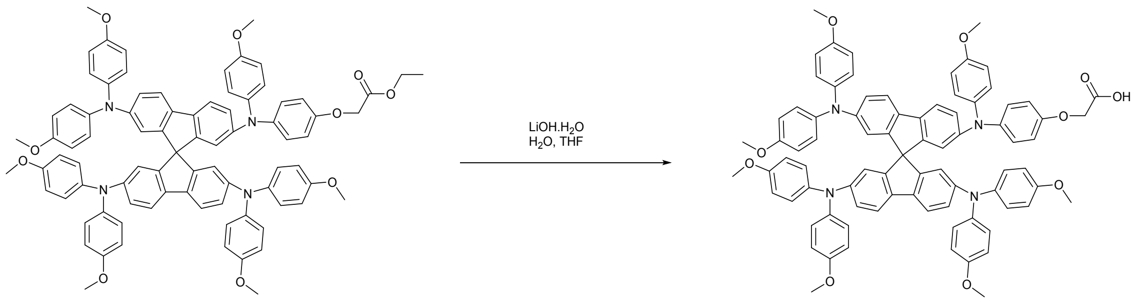

2 Spiro-Acid

Compound **2** (0.400 g, 0.308 mmol) was dissolved in tetrahydrofuran (40 mL). To this mixture, a solution of lithium hydroxide monohydrate (18 mg, 0.462 mmol) in water (4 mL) was added and the resulting mixture was left to stir at room temperature for 2 hours. Then, an aqueous solution of 5% hydrochloric acid was added drop-wise, until pH 1 was reached. The mixture was then diluted with water (150 mL) and the organic part was extracted with ethyl acetate (3 × 100 mL). The combined organic extracts were washed with brine (2 × 150 mL), dried over magnesium

sulfate, filtered, and concentrated in vacuo. The residue was dissolved in a minimum amount of ethyl acetate and added drop wise to hexane (150 mL) while stirring. The precipitate was collected by vacuum filtration, washed with more hexane, and dried under vacuum, affording **Spiro-Acid** (0.285 g, 73%) as an off-white solid. mp > 300 °C. R_f = 0.2 (ethyl acetate:methanol = 9:1). ^{1}H-NMR (C_6D_6, 500 MHz) δ_H 7.15 – 6.88 (28H, m), 6.77 – 6.65 (16H, m), 4.11 (2H, s), 3.31 – 3.24 (21H, m). ^{13}C-NMR (C_6D_6, 125 MHz) δ_C 155.96, 150.84, 148.30, 148.22, 141.97, 135.84, 135.70, 125.88, 125.04, 122.93, 120.74, 118.80, 118.24, 115.93, 114.98, 66.48, 55.00, 54.96, 54.94. HRMS (ESI) m/z: $[M+H]^+$ Calcd for $C_{82}H_{68}N_4O_{10}$ 1269.5009; found 1269.4980.

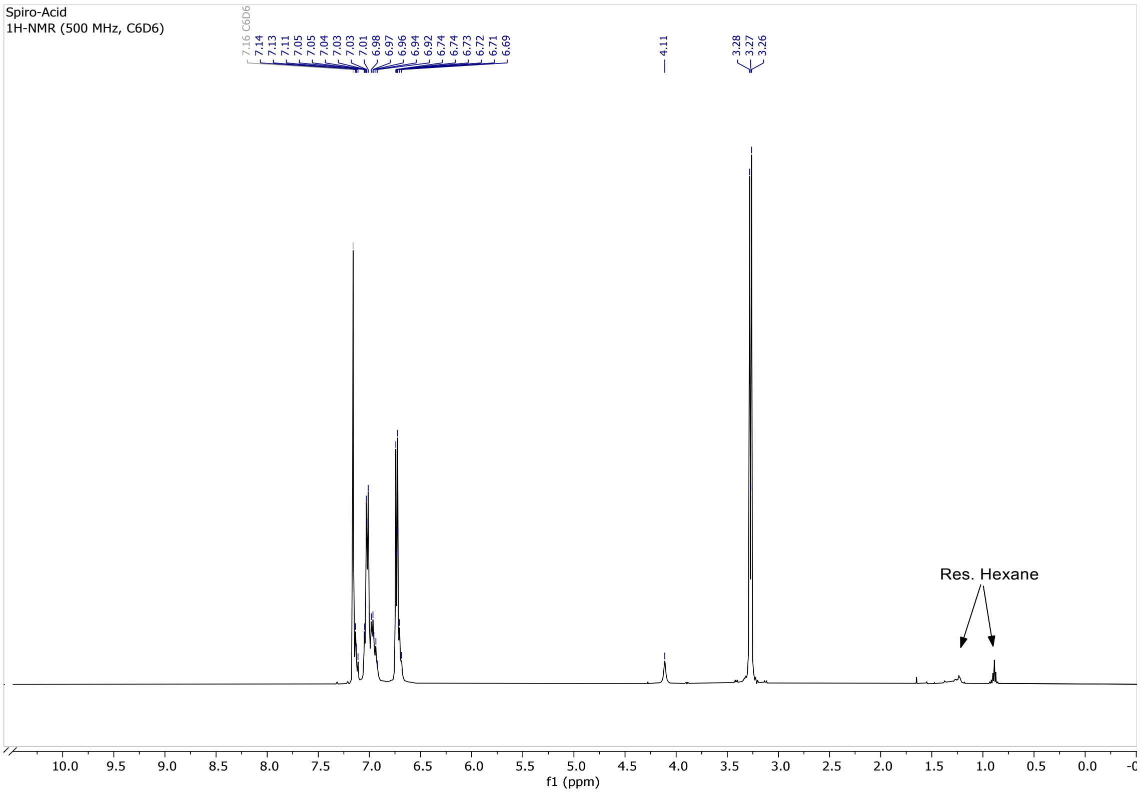

^{1}H NMR of **Spiro Acid**

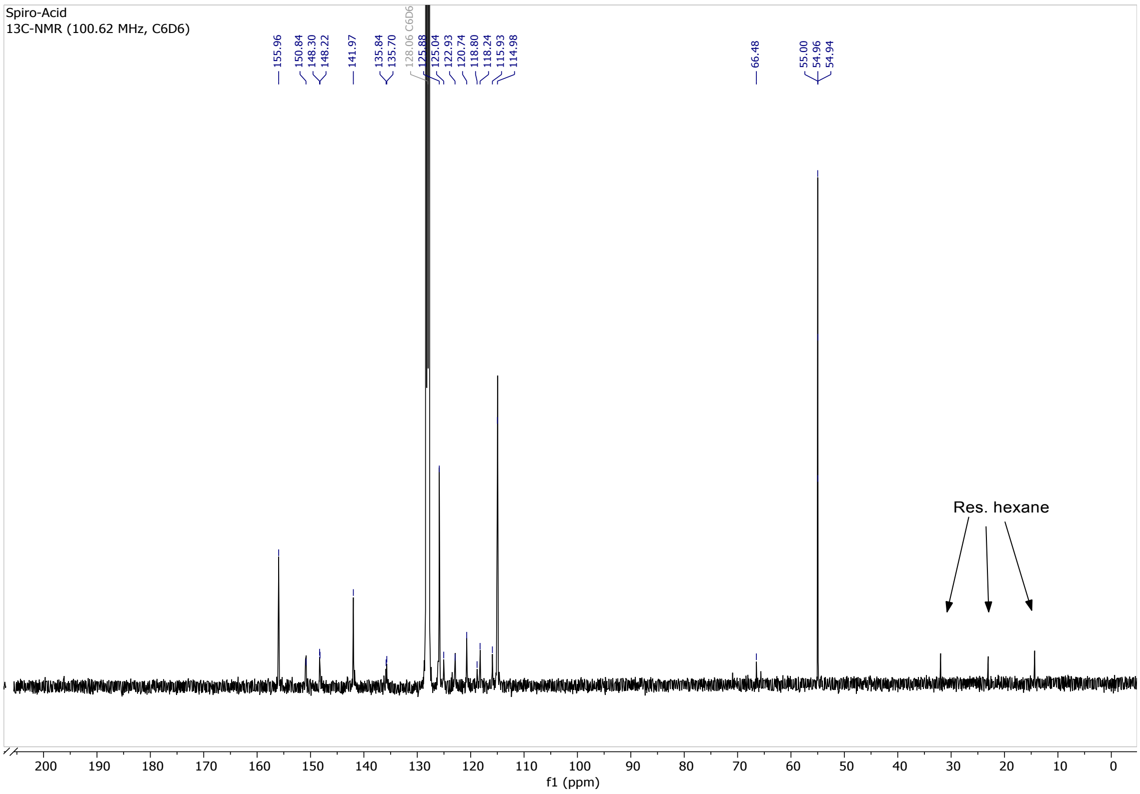

^{13}C NMR of **Spiro Acid**

Custom Workflow Report

Agilent | Trusted Answers

Compound Chromatograms (overlaid)

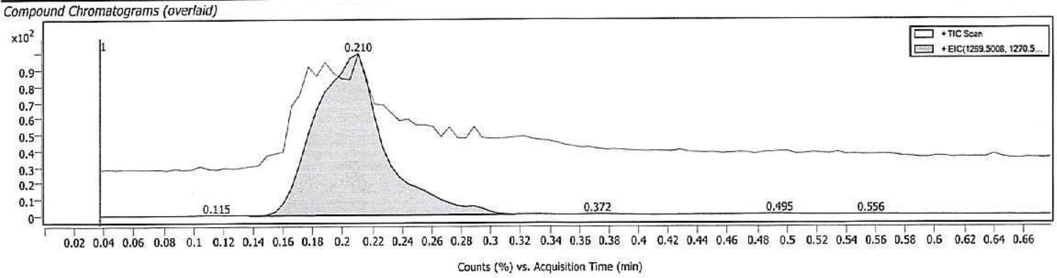

Compound Chromatogram(s)

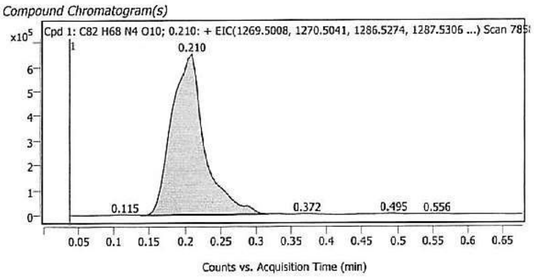

Compound Spectra (overlaid)

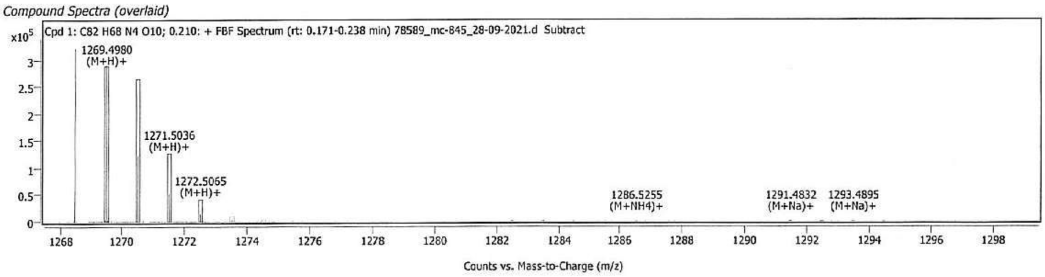

Spectrum Peaks

m/z	Z	Abund	Diff (ppm)	Height %	Height % (Calc)	Ion Species	Formula
1269.4980	1	291149	-2.22	100.00	100.00	(M+H)+	C82H68N4O10
1270.5008	1	124445	-2.63	42.74	91.32	(M+H)+	C82H68N4O10
1271.5036	1	53652	-2.92	18.43	43.27	(M+H)+	C82H68N4O10
1272.5065	1	14843	-3.01	5.10	14.13	(M+H)+	C82H68N4O10
1286.5255	1	128	-1.47	100.00	100.00	(M+NH4)+	C82H68N4O10
1291.4832	1	997	0.32	90.95	100.00	(M+Na)+	C82H68N4O10
1292.4827	1	1096	-2.58	100.00	91.31	(M+Na)+	C82H68N4O10
1293.4895	1	554	0.22	50.52	43.26	(M+Na)+	C82H68N4O10
1294.4907	1	149	-1.25	13.63	14.13	(M+Na)+	C82H68N4O10

Compound Spectra

Thermal analysis of ***Spiro-Acid***

The thermogravimetric analysis (TGA) was performed on a TA Instruments TGA 5500 using a platinum pan, with the temperature ranging from 30 °C to 600 °C, at a rate of 10 °C/min. The differential scanning calorimetry (DSC) was run under N_2 on a TA Instruments DSC 25, using an aluminium pan. The sample was heated from 30 °C to 368 °C, at a rate of 10 °C/min.

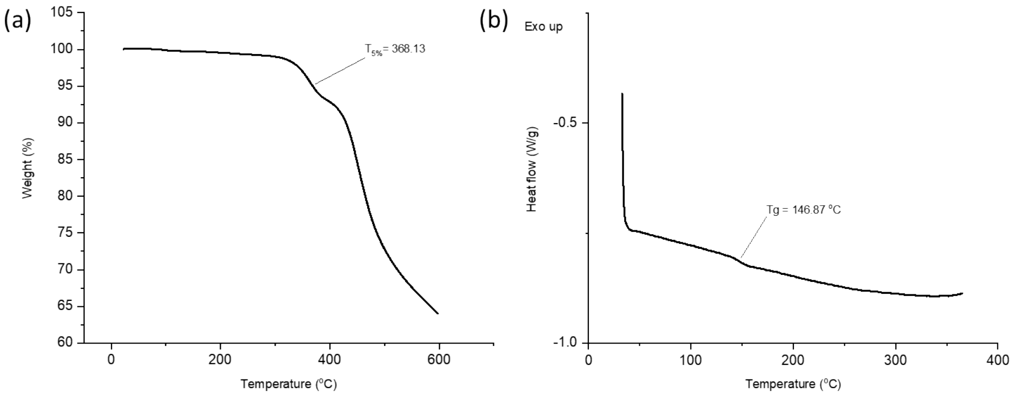

Figure S1. (a) Thermogravimetric analysis of **Spiro-Acid.** $T_{5\%}$ indicates the temperature at which a 5% weight loss is recorded. (b) Differential scanning calorimetry analysis of **Spiro-Acid**. A glass transition is noted at T = Tg.

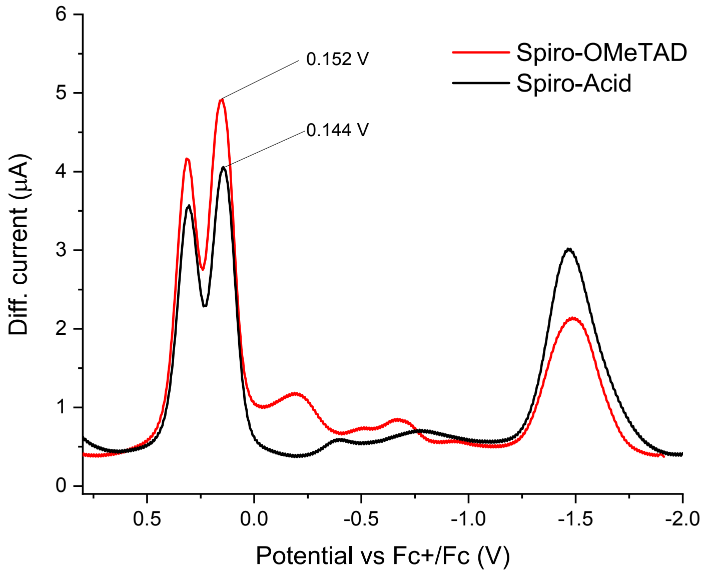

Figure S2. Square wave voltammetry of **Spiro-OMeTAD** and **Spiro-Acid** in solutions of DMF ($C = 5 \times 10^{-4}$ M). Experimental conditions: Pt disc working electrode, Pt wire counter electrode, Ag wire pseudo-reference electrode, Bu_4NPF_6 0.1 M electrolyte. Ferrocene was used as an external standard and potentials referred to the Fc^+/Fc half-wave potential.

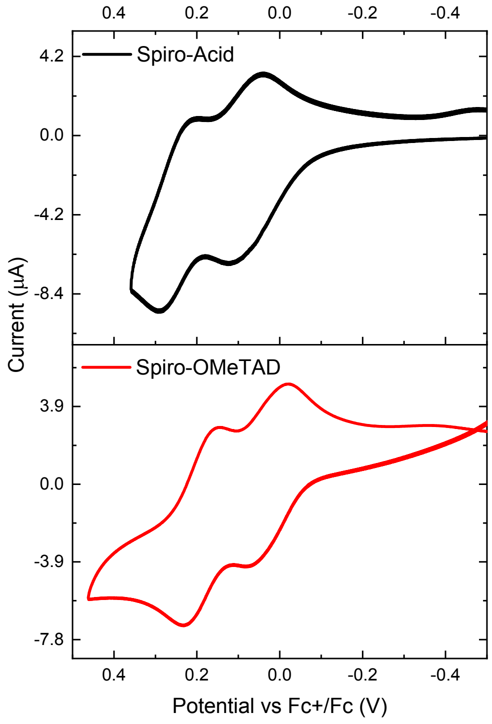

Figure S3. Cyclic voltammetry of **Spiro-OMeTAD** and **Spiro-Acid** in solutions of DMF ($C = 5 \times 10^{-4}$ M). Experimental conditions: Pt disc working electrode, Pt wire counter electrode, Ag wire

pseudo-reference electrode, Bu_4NPF_6 0.1 M electrolyte. Ferrocene was used as an external standard and potentials referred to the Fc^+/Fc half-wave potential.

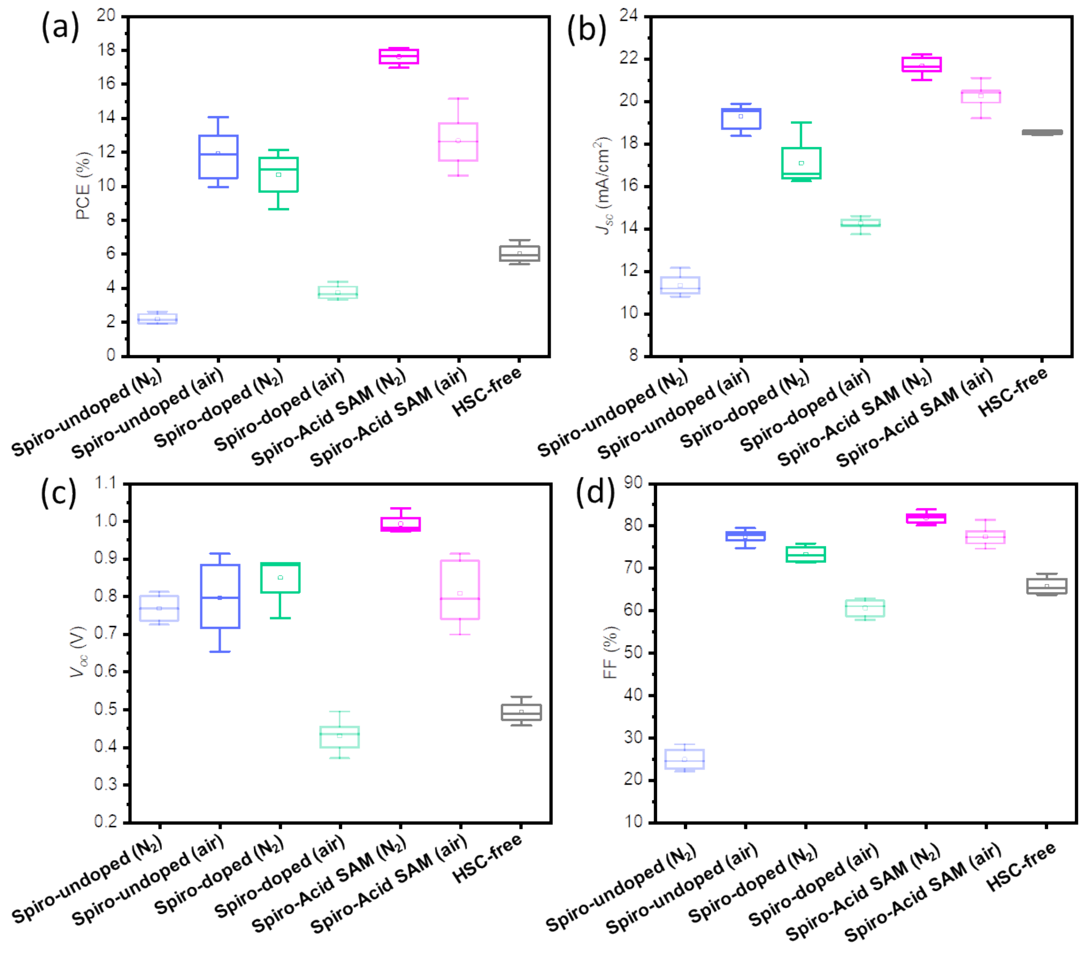

Figure S4. Comparison of Device performance statistics with different HSCs prepared in N_2 or air.

Table S1. Average device parameters of different HSCs prepared in N_2 or air.

HSCs	J_{SC} (mA/cm^2)	V_{OC} (V)	FF (%)	PCE (%)
**Spiro-undoped** (N_2)	11.29±0.59	0.767±0.040	24.7±2.8	2.15±0.33
**Spiro-undoped** (air)	19.28±0.55	0.797±0.099	77.5±1.5	11.92±1.57
**Spiro-doped** (N_2)	17.10±1.28	0.851±0.071	73.3±2.1	10.69±1.48
**Spiro-doped** (air)	14.23±0.36	0.428±0.040	60.5±2.0	3.69±0.41
**Spiro-Acid** (N_2)	21.66±0.43	0.993±0.024	82.0±1.4	17.63±0.46
**Spiro-Acid** (air)	20.22±0.56	0.807±0.083	77.4±2.2	12.65±1.54
HSC-free	18.54±0.10	0.493±0.032	65.8±2.2	6.03±0.60

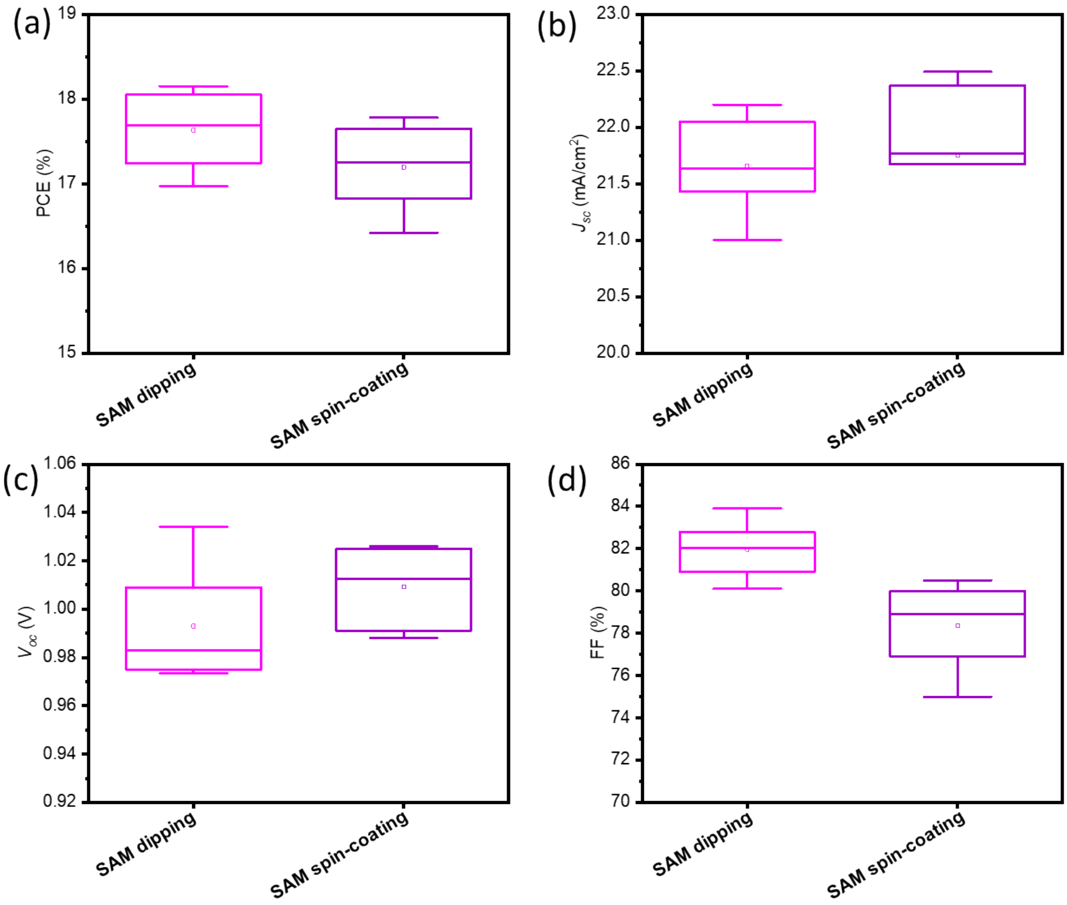

Figure S5. Device performance statistics with **Spiro-Acid** SAMs formed by dipping and spin-coating processes.

Table S2. Average device parameters of **Spiro-Acid** SAMs formed by dipping and spin-coating.

Deposition process	J_{SC} (mA/cm^2)	V_{OC} (V)	FF (%)	PCE (%)
Dipping	21.66±0.43	0.993±0.024	82.0±1.4	17.63±0.46
Spin-coating	21.75±0.73	1.009±0.016	78.4±2.1	17.20±0.57

Table S3. Summary of the device parameters with **undoped Spiro-OMeTAD** based iPSCs from the literature and our work.

HSCs	Deposition methods	J_{SC} (mA/cm^2)	V_{OC} (V)	FF (%)	PCE (%)	HI (%)	Reference
**Undoped Spiro-OMeTAD**	Spin-coating	18.9	0.804	63	9.5	15.79	[2]
**Undoped Spiro-OMeTAD**	Spin-coating	19.7	0.979	76	14.7	5.44	[3]
**Undoped Spiro-OMeTAD**	Spin-coating	22.03	0.99	73	15.92	5.78	[4]
**Undoped Spiro-OMeTAD**	Spin-coating	19.53	0.914	78.8	14.07	10.87	This work
**Spiro-Acid** SAM	Dipping	22.20	0.990	82.6	18.15	2.48	This work

HI: Hysteresis Index. Note that our work shows the highest efficiency with smallest hysteresis (negligible) among the **Spiro-OMeTAD** based p-i-n devices.

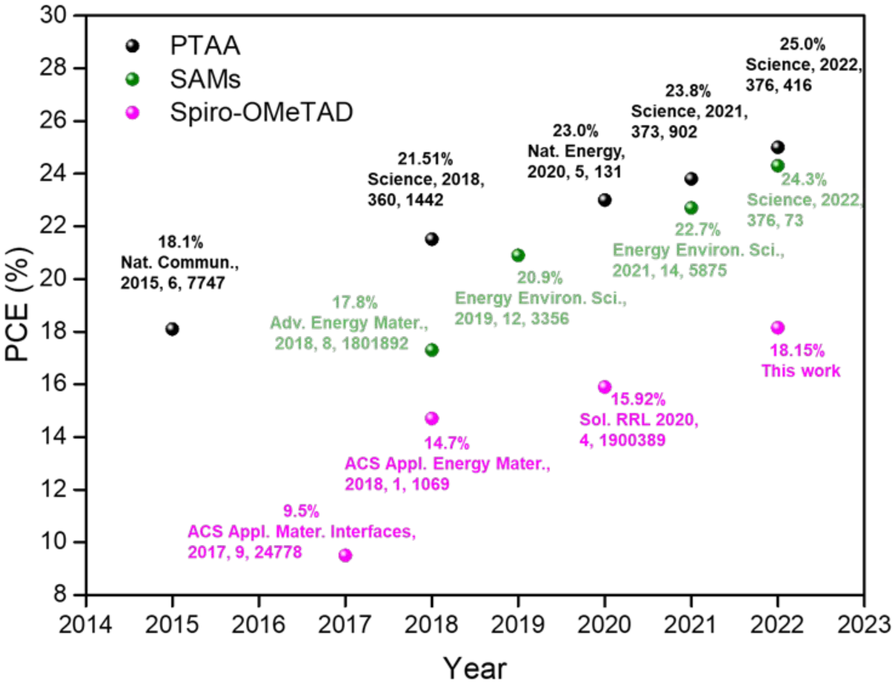

Figure S6. The evolution of efficiency in inverted perovskite solar cells based on **PTAA,** SAMs and **Spiro-OMeTAD** (note that our work is based on $Cs_{0.05}(FA_{0.85}MA_{0.15})_{0.95}Pb(I_{0.85}Br_{0.15})_3$ perovskite without bulk and interfacial defect passivation).

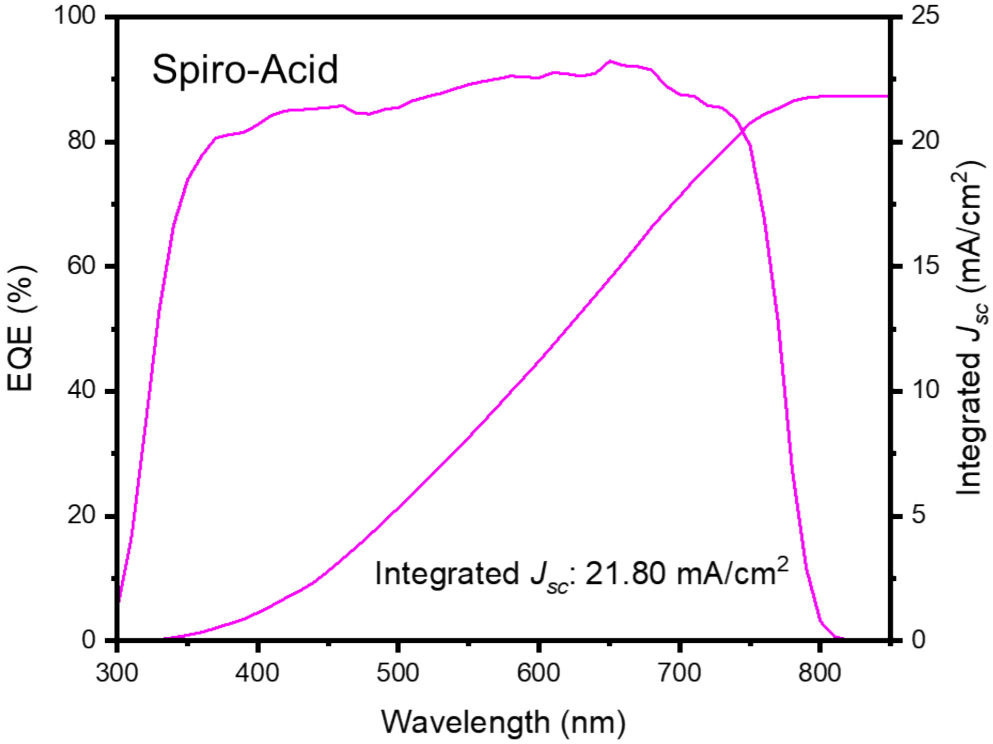

Figure S7. External quantum efficiency (EQE) curve of the best **Spiro-Acid** SAM solar cell and the corresponding integrated J_{SC} from EQE.

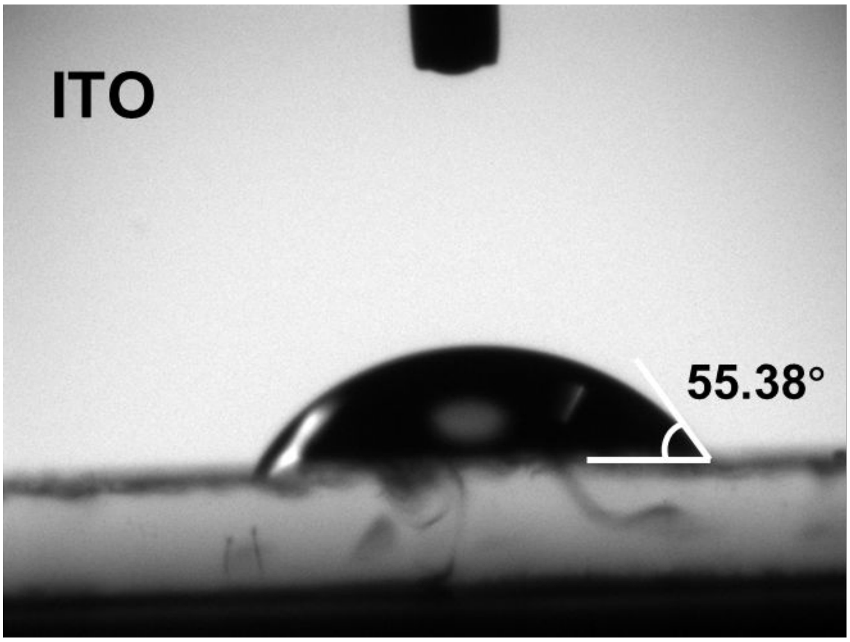

Figure S8. Contact angle of bare ITO with UV-Ozone treatment.

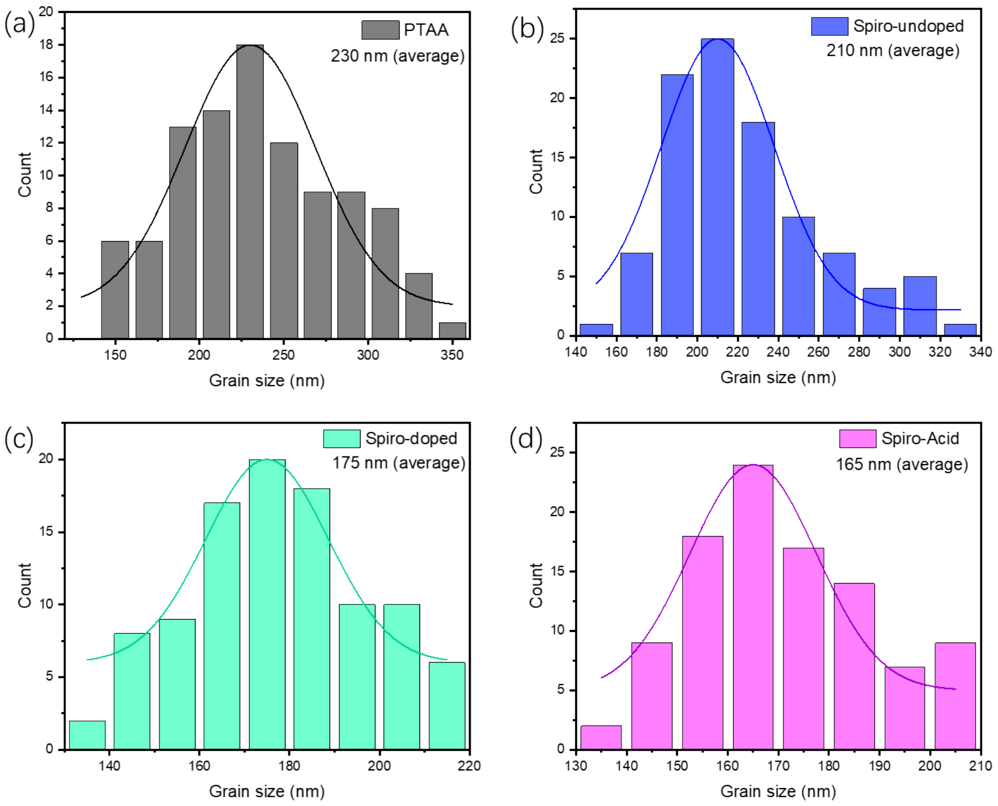

Figure S9. The average grain sizes of perovskite grown on (a) **PTAA**, (b) **Spiro-undoped**, (c) **Spiro-doped** and (d) **Spiro-Acid**.

Table S4. Decay fitting parameters determined from the TRPL curves.

Samples	A_1	τ_1	A_2	τ_2	Counts[a]
ITO/PVK	0.95	18.7	0.87	251.6	2513
ITO/**PTAA**/PVK	5.67	9.9	0.98	75.8	378
ITO/**Spiro-undoped**/PVK	3.35	12.0	0.89	131.8	868
ITO/**Spiro-doped**/PVK	2.24	22.4	0.50	111.4	969
ITO/**Spiro-Acid**/PVK	2.26	12.3	1.02	99.3	538

[a] Counts are from the calculation of collected counts within a fixed 300 s acquisition time.

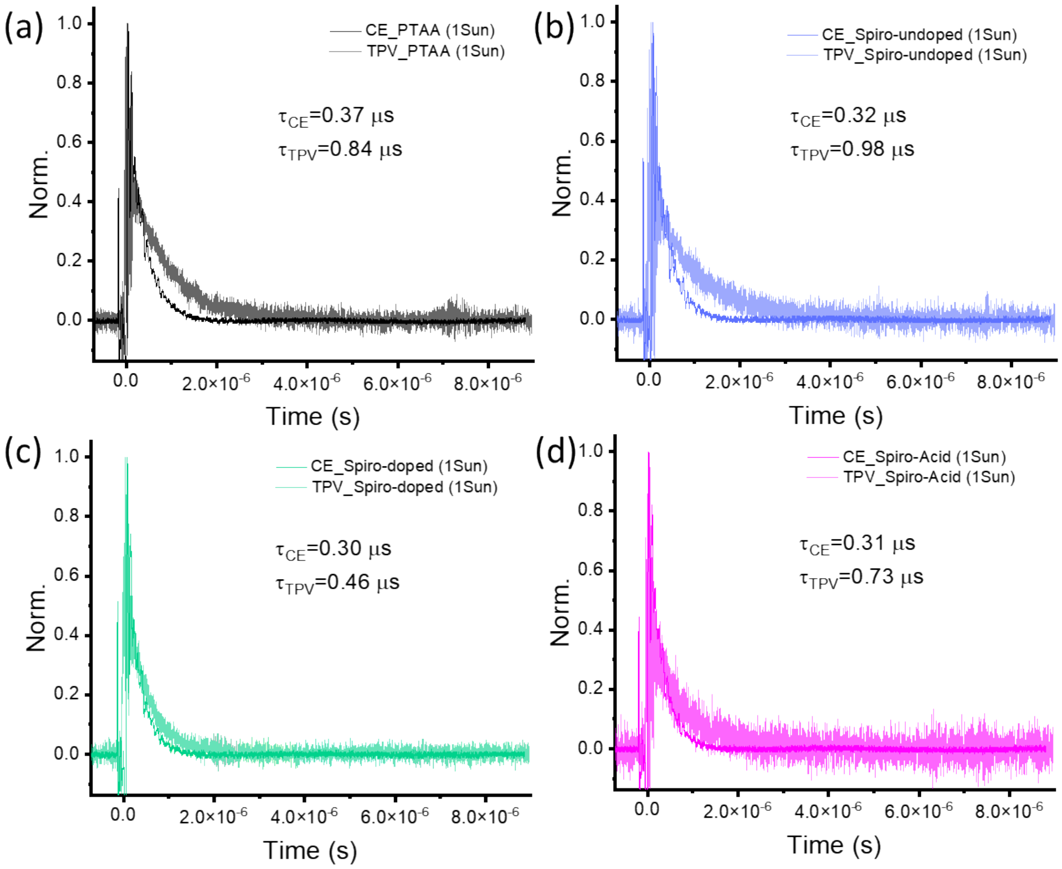

Figure S10. Comparison of the normalized CE and TPV decays measured at 1 Sun conditions for the different hole selective contacts used in this work.

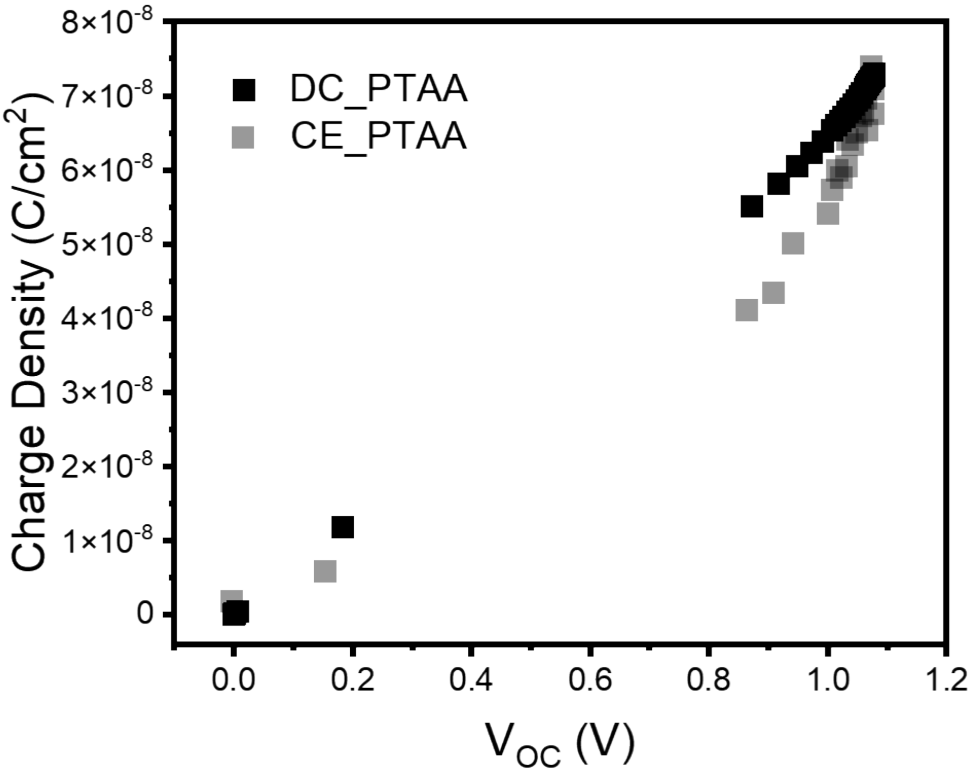

Figure S11. Comparison between the charge density measured by using charge extraction (CE) and differential capacitance (DC) for a device using **PTAA** as hole selective contact.

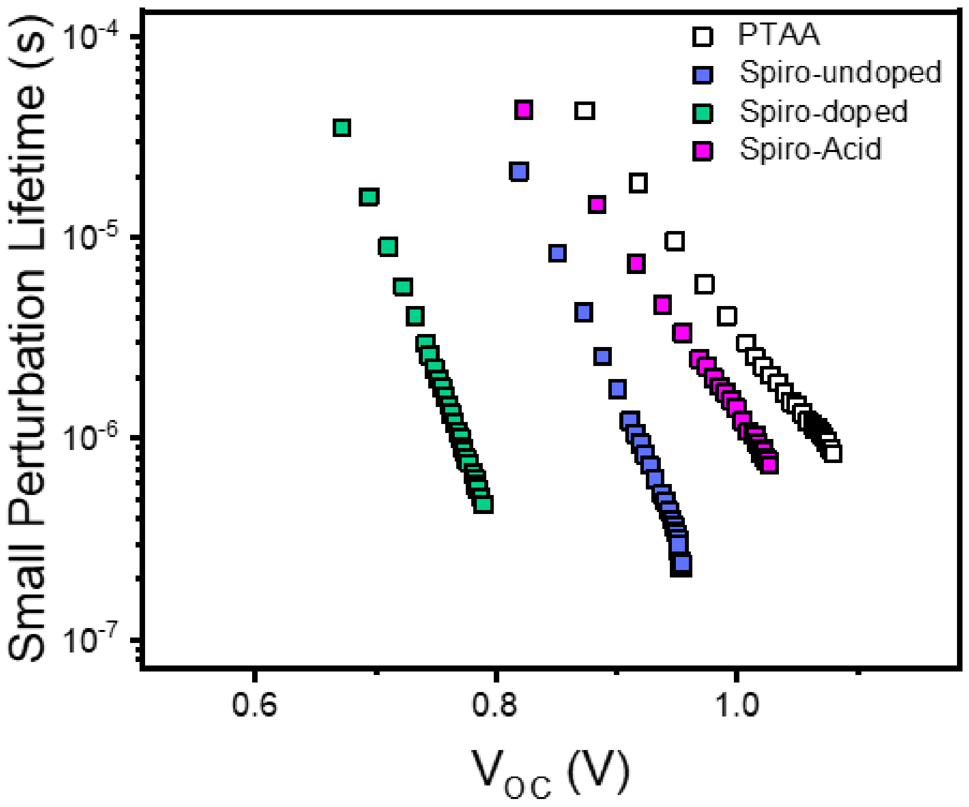

Figure S12. Small perturbation lifetime as a function of Voltage at high light intensities.

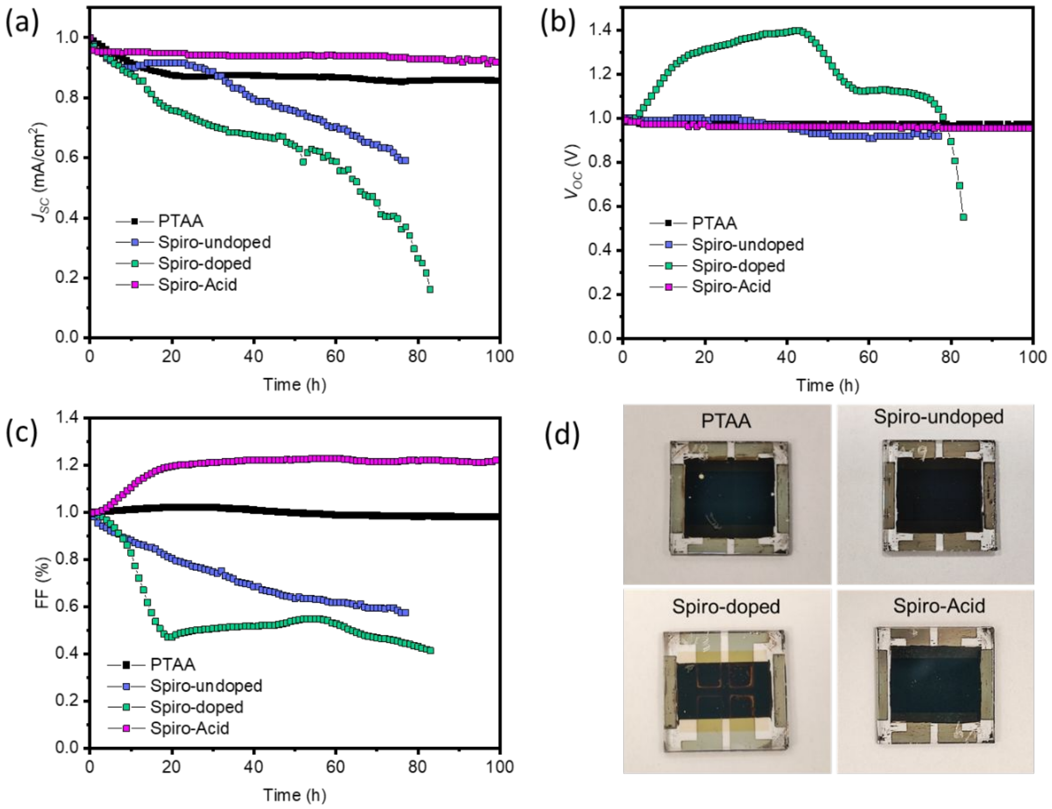

Figure S13. Long-term continuous illumination of PSCs based on different HSCs (a) J_{SC}, (b) V_{OC}, (c) FF, and (d) photos of the devices after illuminating.

References

(1) Cariello, M.; Pant, N.; Harkiss, A. H.; Tracey, F. M.; Cameron, J.; Skabara, P. J.; Holliman, P. J.; Cooke, G. Synthesis of SOT-OH and Its Application as a Building Block for the Synthesis of New Dimeric and Trimeric Spiro-OMeTAD Materials. **2022**. https://doi.org/10.1039/d2me00038e.

(2) Grisorio, R.; Iacobellis, R.; Listorti, A.; De Marco, L.; Cipolla, M. P.; Manca, M.; Rizzo, A.; Abate, A.; Gigli, G.; Suranna, G. P. Rational Design of Molecular Hole-Transporting Materials for Perovskite Solar Cells: Direct versus Inverted Device Configurations. *ACS Appl. Mater. Interfaces* **2017**, *9* (29), 24778–24787. https://doi.org/10.1021/acsami.7b05484.

(3) Iacobellis, R.; Masi, S.; Rizzo, A.; Grisorio, R.; Ambrico, M.; Colella, S.; Ambrico, P. F.; Suranna, G. P.; Listorti, A.; De Marco, L. Addressing the Function of Easily Synthesized Hole Transporters in Direct and Inverted Perovskite Solar Cells. *ACS Appl. Energy Mater.* **2018**, *1* (3), 1069–1076. https://doi.org/10.1021/acsaem.7b00208.

(4) Wang, C.; Hu, J.; Li, C.; Qiu, S.; Liu, X.; Zeng, L.; Liu, C.; Mai, Y.; Guo, F. Spiro-Linked Molecular Hole-Transport Materials for Highly Efficient Inverted Perovskite Solar Cells. *Sol. RRL* **2020**, *4* (3), 1–7. https://doi.org/10.1002/solr.201900389.
